# Attenuation of Hedgehog Acyltransferase-Catalyzed Sonic Hedgehog Palmitoylation Causes Reduced Signaling, Proliferation and Invasiveness of Human Carcinoma Cells

**DOI:** 10.1371/journal.pone.0089899

**Published:** 2014-03-07

**Authors:** Antonios D. Konitsiotis, Shu-Chun Chang, Biljana Jovanović, Paulina Ciepla, Naoko Masumoto, Christopher P. Palmer, Edward W. Tate, John R. Couchman, Anthony I. Magee

**Affiliations:** 1 Molecular Medicine Section, National Heart & Lung Institute, Imperial College London, London, United Kingdom; 2 Department of Chemistry, Imperial College London, London, United Kingdom; 3 Institute of Chemical Biology, Imperial College London, London, United Kingdom; 4 Institute for Health Research and Policy, London Metropolitan University, London, United Kingdom; 5 Department of Biomedical Sciences, University of Copenhagen, Copenhagen, Denmark; Ecole Polytechnique Federale de Lausanne, Switzerland

## Abstract

Overexpression of Hedgehog family proteins contributes to the aetiology of many cancers. To be highly active, Hedgehog proteins must be palmitoylated at their N-terminus by the MBOAT family multispanning membrane enzyme Hedgehog acyltransferase (Hhat). In a pancreatic ductal adenocarcinoma (PDAC) cell line PANC-1 and transfected HEK293a cells Hhat localized to the endoplasmic reticulum. siRNA knockdown showed that Hhat is required for Sonic hedgehog (Shh) palmitoylation, for its assembly into high molecular weight extracellular complexes and for functional activity. Hhat knockdown inhibited Hh autocrine and juxtacrine signaling, and inhibited PDAC cell growth and invasiveness *in vitro*. In addition, Hhat knockdown in a HEK293a cell line constitutively expressing Shh and A549 human non-small cell lung cancer cells inhibited their ability to signal in a juxtacrine/paracrine fashion to the reporter cell lines C3H10T1/2 and Shh-Light2. Our data identify Hhat as a key player in Hh-dependent signaling and tumour cell transformed behaviour.

## Introduction

Hedgehog (Hh) proteins are an important class of secreted intercellular signaling molecules. In mammals, the three Hh homologues, Sonic (Shh), Indian (Ihh), and Desert (Dhh) Hedgehog, play crucial roles in regulation of embryonic development of several organs, including pancreas, digestive system, heart, vascular system and lung. During development, differentiation and tumourigenesis, targets of Hh signaling are involved in cell adhesion, signal transduction, cell cycle, apoptosis and angiogenesis [1]. In adults, Hh signaling is minimal in many differentiated tissues, with the exception of stem cells and thymocytes [2]. However, Hh signaling is aberrantly reactivated in ∼70% of pancreatic ductal adenocarcinomas (PDAC; [7]) as well as many other tumours. Abnormal Hh signaling plays important roles in the growth of many cancer cell types including pancreatic, digestive tract, prostate, breast and lung cancers (small cell lung cancer, SCLC, and non-small cell lung cancer, NSCLC), squamous cell and basal cell carcinomas, gliomas, medulloblastomas and myeloid leukaemias [3]–[6]. Some of these are amongst the most intractable tumors for which no effective therapies exist. Importantly, overexpression of Hh ligands, rather than mutations in Hh pathway components, contributes through autocrine, paracrine or juxtacrine signaling to pathway hyperactivation in PDAC [7], [8], breast [4], [5] and lung [9], [10] cancers. Thus, blocking production of active Shh should downregulate its function and mitigate stimulatory effects on cell growth. Supporting this, treatment with cyclopamine, a specific inhibitor of the positive transducer of Hh signaling, Smoothened (Smo), reduces the viability or proliferation of several cancer cell types (see refs above). Smo antagonists are under development as cancer therapeutics and are in clinical trials for multiple cancers (Hedgehog pathway inhibitors, ClinicalTrials.gov., National Institutes of Health, June 2012).

Hh proteins are secreted through the secretory pathway via the endoplasmic reticulum (ER) and Golgi complex, but an unusual feature is their post-translational modification by addition of a fatty acid (palmitate, Pal) and cholesterol to the protein [11]. These modifications are essential for the controlled extracellular spread of Hhs to target cells and their biological activity. During intracellular transport, the ∼45 kDa Shh precursor is palmitoylated on its conserved N-terminal cysteine residue. Concomitantly, a ∼18 kDa N-terminal fragment (Shh-N) and a ∼25 kDa C-terminal fragment (Shh-C) are generated by intein-like autocatalytic cleavage catalyzed by Shh-C and, concurrently, cholesterol is covalently attached to the C-terminus of Shh-N to form the mature active Shh-Np [12]. In mammals, Shh palmitoylation is crucial for its biological activity; e.g. removal of the palmitoylation site abrogates the ability to induce differentiation of E11 telencephalic neurons during rodent ventral forebrain formation [13]. Palmitoylation also plays a major role in guiding modified Hh proteins to specific membrane domains [14]. The cholesterol attached to Shh-Np, on the other hand, enhances its affinity for cell membranes and regulates its cell surface distribution (e.g. to membrane microdomains), and also affects its extracellular range and concentration gradient from the producing cell. Dual lipidation of Shh improves membrane affinity, and is necessary for formation of high molecular weight complexes with heparan sulphate proteoglycans (HSPGs; [12]) which enhances signaling and *in vivo* activity [14], [15]. These complexes assemble during transport to the cell surface and are of ill-defined composition, but have been reported to contain Hh, HSPGs and lipoproteins [16]. However, whether the released multimeric form of Shh still contains its lipid modifications and is a hetero or homomultimer is a matter of controversy [17]. Indeed, lipidation of Shh has been reported to be necessary for the cleavage and release of active Shh multimers during which the lipidated termini are removed [18].

Hh signaling in receiving cells is regulated by Patched (Ptch) and Smo [5]. In the absence of Hhs, the heptahelical protein Smo is inhibited by the Ptch Hh receptors. Hhs bind to Ptch and relieve inhibition of Smo, allowing activation of downstream signaling, ultimately via Gli transcription factors in vertebrates. The exact mechanisms of these inhibitory interactions are unclear; current models suggest Smo is retained intracellularly in the absence of Hh and translocates to the plasma membrane when Ptch binds Hh, localizing in the primary cilium where activation of Gli proteins occurs [19]. Targets of Hh signaling include several pathway components, e.g. Ptch, Gli, and upregulation of their expression can be used to assay Hh pathway activity.

Hedgehog acyltransferase (Hhat) is responsible for palmitoylation of Hhs [20]–[22] (note Hhat was previously designated skn, ski, sit and rasp). It is a member of the MBOAT family of membrane-bound acyltransferases, predicted to contain between 8–12 transmembrane domains (Figure S1) [23]. These multispanning transmembrane enzymes usually catalyze the addition of a fatty acid to membrane-embedded substrates such as lipids [24]. Three MBOAT family members acylate protein substrates: Hhat, Porcupine (Porc; substrates Wg/Wnt proteins) and ghrelin O-acyltransferase (GOAT; octanoylates the substrate ghrelin, an appetite-controlling peptide) [25], [26]. MBOAT proteins contain a characteristic histidine in one transmembrane domain, being conserved in most family members and thought to be involved in their acyltransferase activity based on mutational studies.

We show here, using fluorescent protein fusions and epitope-tagged Hhat proteins, that Hhat predominantly localizes in the ER. Hhat knockdown (KD) in the PANC1 PDAC cell line reduces palmitoylation of Shh, prevents its assembly into multimeric complexes, causes suppression of signaling through the Hh pathway, and reduces growth and invasiveness. Growth inhibition by Hhat KD was also shown for A818 PDAC cells. In addition, Hhat KD in HEK293a cells constitutively expressing Shh and A549 human NSCLC cells inhibited their juxtacrine/paracrine signaling. We demonstrate an important role for Hhat in PDAC and other tumour cells and provide evidence that Hhat inhibition is a target for tumour growth suppression.

## Materials and Methods

### Cell culture and siRNA transfection

Human pancreatic ductal adenocarcinoma PANC1 cells (ATCC, CRL-1469) were maintained in Dulbecco's modified Eagle's medium (DMEM) supplemented with 8% fetal bovine serum (FBS). A818-1 cells [50] were a gift of Mr. Hemant Kocher (Barts Cancer Institute, London) cells were grown in DMEM plus 10% FBS, plus 100 u/ml penicillin and 100 µg/ml streptomycin. The human embryonic kidney 293a (HEK293a) line was generously provided by Dr. Birgit Leitinger (Imperial College London). HEK293a cells were maintained in DMEM supplemented with 8% FBS. Human A549 non-small cell lung cancer cells were a kind gift of Prof. Simak Ali (Department of Surgery and Cancer, Imperial College London) and were maintained in DMEM supplemented with 10% FBS. Shh-Light2 cells [38] were a kind gift from Drs. Marta Swierczinska and Suzanne Eaton (Max Planck Institute for Cell Biology and Genetics, Dresden) and were grown in DMEM supplemented with 10% FBS, 400 µg/ml G418 (geneticin, Sigma) and 150 µg/ml Zeocin (Invitrogen). Mouse C3H10T1/2 osteoblast precursor cells (ATCC CCL-226; [31]) were a kind gift of Dr. Kay Grobe, University of Muenster, Germany. Cells were validated by microsatellite genotyping (STR-PCR based method in May 2013; Public Health England, Salisbury, UK).

Human Hhat siRNA duplex oligomers Hhat-#1 (sense strand 5′-UUAAUCAGGUAUGUGUACAUUCCAGUG-3′) were designed and ordered from MWG Biotech. Mutated Hhat siRNA duplex oligomers (5′-UUAAUCAGGCAUAUGUACGUUCCAGUG-3′) were from Invitrogen. ON-TARGET plus siRNA against human Hhat (5′-AGGACAGUCUGGCCCGAUA-3′; Hhat-#2) and ON-TARGET plus Non-targeting siRNA pool were from Dharmacon (Thermo Scientific Dharmacon; Epsom, UK). Another negative control applied in this study was SilencerR Negative Control #1 siRNA (Hhat-Scr, Ambion).

siRNA transfections were carried out by plating 0.3 million cells per well in a 6-well plate and 6 h later treated with 20 nM siRNA oligomers and 3 µl FuGENE 6 Transfection Reagent (Roche) per reaction. Alternatively, siRNA transfections were carried out using the Metafectene SI reagent (Biontex, Martinsried/Planegg, Germany) according to the manufacturer's instructions. 30 pmol RNA and 1 µl Metafectene SI reagent were diluted in 30 µl 1× Metafectene SI buffer and allowed to complex for 20 min at room temperature (RT) in a well of a 48-well plate. In the meantime, cells were trypsinized and diluted to 0.8×10^5^ cells/ml in complete medium. Suspended cells (250 µl) were added to each well and plates were shaken to ensure even distribution of the cells and reagents. Cells were assayed 72 h post-transfection.

### Immunoblotting

Immunoblotting was carried out 24 h after transfection of HEK293 cells with expression vector for Hhat-EGFP. For SDS-PAGE, 0.4 million cells were harvested and lysed by the addition of loading buffer (45 mM Tris (pH 6.8), 10% glycerol, 1% SDS, 20 mM DTT, 0.01% bromophenol blue) followed by syringing. After electrophoresis, proteins were transferred to nitrocellulose membranes (Millipore, Bedford, MA, USA), blocked at room temperature for 1 h in phosphate-buffered saline (PBS) with 5% skimmed milk, and then incubated with primary antibody for 16 h at 4°C. Goat polyclonal anti-Patched (ab51983) and mouse monoclonal α-Tubulin (DM1A, ab49928) were purchased from Abcam. Rabbit polyclonal anti-GLI-1 (H-300, sc-20687) and rabbit polyclonal anti-Shh (H-160, sc-9024) were purchased from Santa Cruz Biotechnology. Mouse monoclonal anti-GFP was from Roche. Secondary antibodies were horseradish peroxidase (HRP)-conjugated goat anti-mouse immunoglobulin IgG1, HRP-conjugated goat anti-rabbit IgG, and HRP-conjugated donkey anti-goat IgG (used at 1∶20,000; Southern Biotech). Bound immunocomplexes were detected using enhanced chemiluminescence detection reagents (Pierce) and were visualized by exposing the membrane to x-ray film (Fuji Super RX), or with ECL Plus reagent and the Ettan DIGE Imager (GE Healthcare).

### Immunostaining and fluorescence microscopy

PANC1 cells were seeded onto 6-well plates at a density of 1×105 cells/well and transfected with Hhat-EGFP. 48 h after transfection, the cells were fixed with 4% paraformaldehyde (PFA). Alternatively, 6×104 HEK293a cells stably expressing Hhat-V5 were seeded onto glass coverslips in 24-well plates. 24 h after plating out, cells were fixed with 3% PFA in 1× PBS. Imaging was performed using a Zeiss LSM510 laser-scanning confocal microscope in the Imperial College Facility for Imaging by Light Microscopy (FILM). Hhat was visualized with mouse monoclonal anti-V5 IgG2A (1∶200, Invitrogen) followed by Alexa488-conjugated anti-mouse IgG2A secondary antibody. Golgi complex was visualized after staining with mouse monoclonal anti-GM130 IgG1 (1∶600, BD Transduction Laboratories™), and ER with mouse monoclonal anti-protein disulfide isomerase (PDI) IgG1 (1∶100, BD Transduction Laboratories™) or anti-calnexin (AF18, 1∶50, Sigma) respectively, followed by Alexa568 or Alexa633-conjugated anti-mouse IgG1 secondary antibodies.

### Semiquantitative Reverse Transcription-PCR (RT-PCR) and qPCR

Total RNA extraction from cultured cells was done with Trizol (Invitrogen). cDNA was synthesized by random priming from 1 µg of total RNA with SuperScript II Reverse Transcriptase kit (Invitrogen), according to the manufacturer's instructions. Alternatively, the cDNA was synthesized by random priming from 1 µg of total RNA with the GoScript reverse transcriptase kit (Promega). The following primers were used for the subsequent PCR: human GAPDH (sense, 5′-TTCATTGACCTCAACTACAT-3′; antisense, 5′-GTGGCAGTGATGGCATGGAC-3′); human β-actin (sense, 5′-ATGGATGAGGATATCGCTGCG-3′; antisense, 5′-CTAGAAGCATTTGCGGTGCAC-3′); human Shh (sense, 5′-CGCACGGGGACAGCTCGGAAGT-3′; antisense, 5′-CTGCGCGGCCCTCGTAGTGC-3′) [26]; human Ptch (sense, 5′-GGTGGCACAGTCAAGAACA-3′; antisense, 5′-ACCAAGAGCGAGAAATGG-3′) [26t]; human Smo (sense, 5′-TTACCTTCAGCTGCCACTTCTACG-3′; antisense, 5′-GCCTTGGCAATCATCTTGCTCTTC-3′) [27]; human Gli1 (sense, 5′-TTCCTACCAGAGTCCCAAGT-3′; antisense, 5′-CCCTATGTGAAGCCCTATTT-3′) [27].

Polymerase chain reaction (PCR) with Taq DNA Polymerase (Invitrogen) followed the manufacturer's instructions. Cycling conditions were: 30 cycles of 30 seconds at 95°C, 30 seconds at 60°C, and 2 minutes/kb at 72°C. PCR products were resolved by electrophoresis on 1.7% agarose gels and visualized by ethidium bromide staining.

Hhat knockdown in the siRNA-treated cells was further validated by qPCR, for which we used the GoTaq qPCR master mix (Promega). Specifically, cDNA from 15 ng input RNA was used in a 20 µl reaction in a 96-well thermal plate, in triplicate. The plates were sealed and run in an ABI 7500 Fast Real-Time PCR System cycler (Applied Biosystems, Life Technologies). Cycling conditions: 40–45 cycles of 95°C for 45 sec, 60°C for 30 sec. Data were analyzed using the ΔΔCt method for determination of relative gene expression by normalization to an internal control gene (GAPDH) and fold expression change was determined compared to a control sample treated with non-targeting siRNA.

### Cloning and Expression of Recombinant Hhat

Human Hhat cDNA (Accession No: BC117130) was cloned into pEGFP-N3 mammalian expression vector at the XhoI and BamHI sites. Transfections of mammalian expression vectors were carried out using Lipofectamine 2000 (Invitrogen) or FuGENE HD (view section above for details; Promega). HA-Hhat-V5 and Hhat-V5 were created using Gateway® cloning (Invitrogen) into mammalian expression vector pcDNA-DEST40. Entry vectors were made by topoisomerase cloning of PCR inserts into vector pENTR/D-TOPO using pENTR directional TOPO cloning kit. PCR inserts were amplified from human Hhat cDNA (Accession No: BC117130) using forward primer 5′-CACCATGTACCCCTACGACGTGCCCGACTACGCCCTGCCCCGATGGGAACTG-3′ or 5′-CACCATGCTGCCCCGATGG-3′ and reverse primer 5′-GTCCGTGGCGTAGGTCTG-3′.

Entry vector pENTR/D-TOPO containing Hhat constructs and destination vector pcDNA-DEST40 were recombined using LR Clonase II enzyme mix to produce HA-Hhat-V5 or Hhat-V5 expressing constructs. Resulting clones were confirmed by DNA sequencing.

### Click chemistry/*in vitro* palmitoylation

Forty eight hours after siRNA transfection, culture medium was changed to DMEM labeling medium (1 mM sodium pyruvate and 50–100 µM azido-palmitate analogue, 15-hexadecynoic acid YnC15 [28], [29] for 16–36 h. Cells were washed in PBS, and cell extracts were prepared in lysis buffer (1% Triton X-100 and protease inhibitors (complete protease inhibitor cocktail EDTA-free, Roche) in PBS, pH 7.4). Equivalent amounts of cell lysates and medium were immunoprecipitated for Shh separately using 5E1 anti-Shh MAb (purified in this lab from 5E1 hybridoma cells obtained from the Developmental Studies Hybridoma Bank, USA; free of any antimicrobial solution or preservatives that could affect cell viability).

Copper-catalyzed azide-alkyne cycloaddition (CuAAC; click chemistry, [28]–[30]) was carried out directly on protein G-agarose beads using azido-TAMRA-biotin capture reagent [28], [29]. *In vitro* palmitoylation was detected by fluorescence imaging with an Ettan DIGE Imager (GE Healthcare, UK). Protein concentrations were measured using Bio-Rad Protein Assay Reagent (Bio-Rad, Richmond, CA, USA) following the manufacturer's suggested procedure.

### Shh oligomerization assay

Seventy two hours after Hhat siRNA transfection, culture medium was changed to serum-free medium (SFM) for 24 h. Gel filtration analysis of clarified conditioned SFM was performed by fast protein liquid chromatography (ÄKTA Protein Purifier, Amersham Biosciences) using a Superdex200 10/300 GL column (Amersham Biosciences) equilibrated with PBS at 4°C [31]. Eluted fractions were trichloroacetic acid-precipitated and probed for Shh by dot blotting with anti-Shh H-160 (Santa Cruz, sc-9024).

### Matrigel invasion assay

Biocoat Matrigel Invasion Chambers with 8 µm pores in 6-well plates (BD Biosciences) were used for invasion assays [32]. To determine the effect of Hhat knockdown, PANC1 cells were pretreated with Hhat siRNA (described above) for 72 h before they were added to the chamber. PANC1 cells were detached with 5 mM EDTA in PBS, resuspended in serum-free DMEM and added to the upper compartment of the chamber (1×10^5^ cells/well). Conditioned medium (8% FBS) was placed in the lower chamber. After 24 h incubation at 37°C, the cells on the upper surface were completely removed by wiping with a cotton swab, and then the filter was fixed with 100% methanol and stained with crystal violet solution. Cells that had migrated from the upper to the lower side of the filter were photographed and counted with a light microscope (40 fields/filter).

### PANC1 proliferation assay

Proliferation was measured *in vitro* with the vital dye 5(6)-Carboxyfluorescein diacetate *N*-succinimidyl ester (CFSE, Sigma), which is loaded into cells and becomes diluted during subsequent cell divisions. Briefly, after 24 h of synchronization in serum-free medium, cells were transfected with siRNAs (test and controls) or treated with anti-Shh 5E1 blocking antibody (Day 1, 10 µg/ml) and then all conditions were labeled with 2.5 µM CFSE at 37°C for 15 min. For some wells, 5E1 treatment was carried out 72 h after synchronization (Day 4, 10 µg/ml), to mimic the kinetics of RNAi knockdown. After 8 days of culture, cell division was indicated by decreased CFSE fluorescence intensity, as analyzed by flow cytometry. For flow cytometer assessment, PANC1 cells were removed from plates and resuspended in ice-cold FACS buffer (1% FBS and 2 mM EDTA in PBS), then analyzed with a FACScalibur flow cytometer.

### Paracrine Hh signaling assay with C3H10T1/2 cells

A549 or HEK293a-Shh cells were seeded in co-culture with C3H10T1/2 cells in DMEM + 3% FBS at a ratio of 1∶2 of cells of interest to C3H10T1/2 cells. A total of 14,800 cells were seeded per well in a 96-well plate. For Shh pathway inhibition, medium was supplemented with 20 µg/ml Shh blocking monoclonal antibody 5E1 or 5 µM cyclopamine at seeding. For Hhat siRNA KD, A549 or HEK293a-Shh cells were treated for 48 h with 20 µM siRNA Hhat-#2 5′-AGGACAGUCUGGCCCGAUA-3′ (Dharmacon) or a pool of four non-targeting siRNAs (ON-TARGETplus Non-Targeting pool, D-001810-10-05, Dharmacon), then trypsinized and seeded in co-culture with C3H10T1/2 cells in DMEM + 3% FBS as described. After two days of culture, medium was removed, cells were washed in PBS and lysed by gentle shaking on a horizontal shaker on ice for 10 min in 50 µl of ice-cold lysis buffer (PBS pH 7.4, 0.1% (v/v) Triton-X100, Complete Mini EDTA-free protease inhibitor (Roche)). Alkaline phosphatase (ALP) activity was measured using p-nitrophenyl phosphate as substrate. 5 mg p-nitrophenyl phosphate (Sigma) were dissolved in 5 ml ALP reagent buffer (1 M diethanolamine buffer pH 9.8, 0.5 mM MgCl_2_). 200 µl of ALP substrate solution were added per well of lysed cells and the reaction was incubated at room temperature for 1–2 h. Reaction was stopped by addition of 50 µl 3 M NaOH and absorbance was measured at 405 nm.

### Paracrine Hh signaling assay with Shh-Light2 cells

Shh-Light2 cells are an NIH/3T3 cell line commonly used in Shh reporter assays. These cells stably express a firefly luciferase gene under a Gli-inducible promoter, as a reporter of Shh activity, and constitutively express a Renilla luciferase gene in order to normalize the data to cell numbers. 2.5×10^3^ PANC1, 5×10^3^ HEK293a or 5×10^3^ HEK293a-Shh cells were seeded in a 96-well plate in co-culture with 10×10^3^ Shh-Light2 cells in DMEM supplemented with 3% FBS; cells were cultured for 72 h. Firefly luciferase expression was measured using the Dual-Luciferase reporter system (Promega) according to the manufacturer's instructions. Briefly, cells were lysed in 25 µl of passive lysis buffer, at RT for 15 min with shaking. 5 µl of lysate were then added to a single well of a black 96-well assay plate (Corning). 20 µl of luciferase assay reagent II were then added, and firefly luminescence measured for 10 sec. After the measurement, firefly luciferase was quenched and Renilla luciferase measured by the addition of 20 µl Stop&Glo reagent. Luminescence was measured in a FLUOstar Optima plate reader (BMG Labtech, Aylesbury, UK) and data were normalized to the Renilla luciferase readout. For Hhat siRNA KD, 2×10^4^ PANC1 cells were transfected with Hhat-9 siRNA as described above. 48 h post-siRNA transfection the medium was exchanged for DMEM supplemented with 3% FBS containing 1×10^5^ Shh-Light2 cells. Cells were co-cultured for 72 h, and then lysed in 50 µl of passive lysis buffer and luciferase measured as described above.

### Statistical analysis

All experiments were replicated at least three times, and statistical significance was measured by using the two-tailed t test. A p value of less than 0.05 was considered to indicate statistical significance. All signals from immunoblots and dot blots were quantified using Scion Image software.

## Results

### Intracellular localization of Hhat to ER

**Figure 1:**
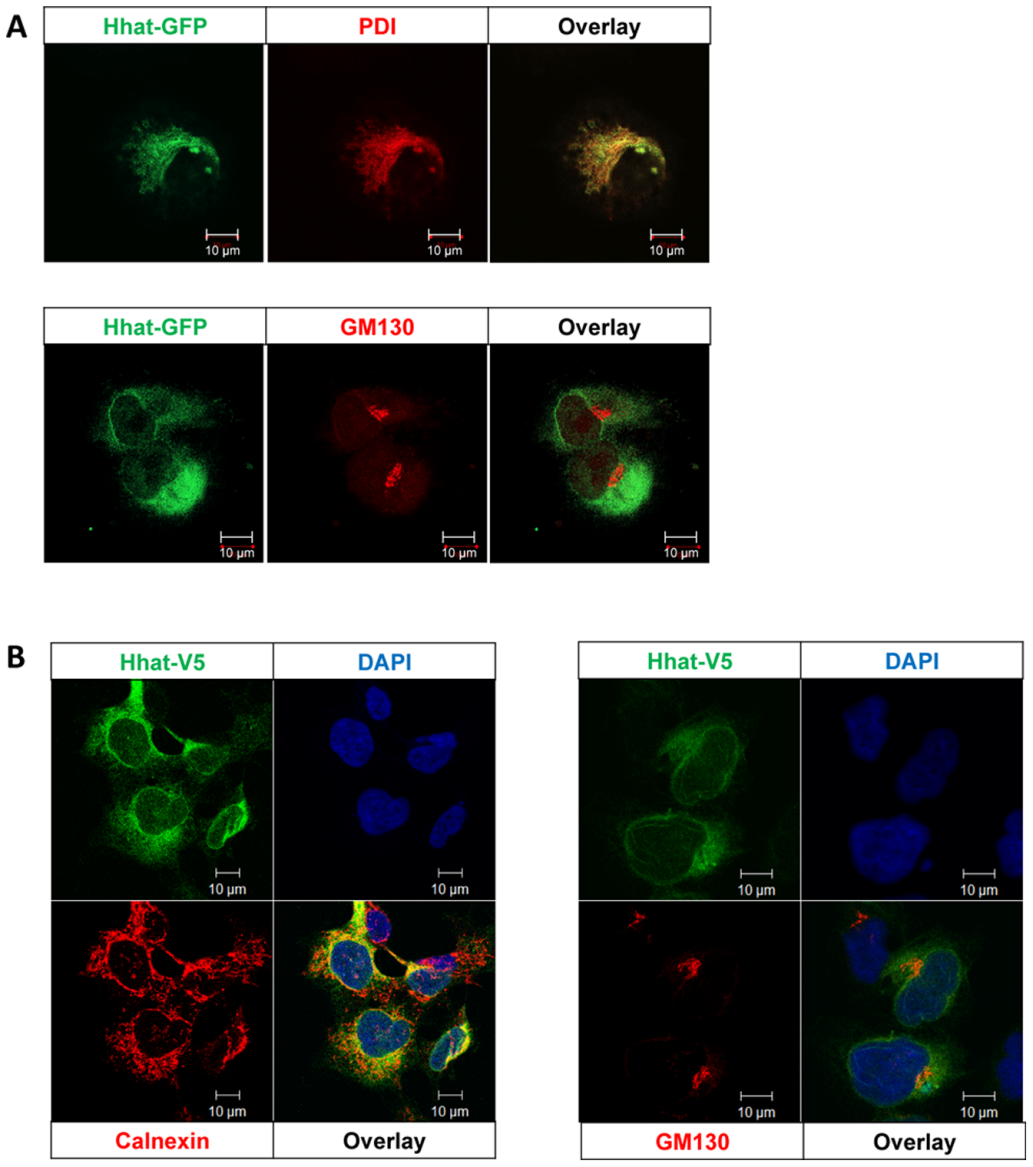
Localization of Hhat in PANC1 cells. A. Localization of Hhat in PANC1 cells was assessed using Hhat-EGFP transfection and confocal microscopy combined with immunofluorescence localization of ER (PDI) and Golgi (GM130). B. HEK293a Hhat-V5 stable cells were co-stained for the V5 epitope with ER (Calnexin) or Golgi (GM130) and nuclei (DAPI). The data show that both Hhat-EGFP and Hhat-V5 localize primarily in ER with little if any in Golgi apparatus. Scale bar = 10 µm.

To determine the intracellular localization of Hhat in PANC1 cells, they were transfected with Hhat-EGFP followed by laser scanning confocal microscopy; anti-GM130 and anti-PDI or anti-calnexin were used to localize Golgi complex and ER respectively by immunofluorescence confocal microscopy. Hhat-EGFP was primarily in the ER (Figure 1A; Pearson correlation coefficient = 0.67±0.20). Z-sections taken every 0.5 µm through the cell showed insignificant, if any, Hhat within the Golgi complex (Figure 1A; Pearson correlation coefficient = 0.12±0.11). Similar results were obtained by staining for Hhat-V5 in stably transfected HEK293a cells (Figure 1B). In this case, the Pearson correlation coefficient between Hhat-V5 and the ER was 0.75±0.06, and between Hhat-V5 and Golgi was 0.28±0.08. Our data differ somewhat from those of Resh and colleagues who reported substantial localization to the Golgi complex as well as ER [33]. ER localization of Hhat was the predominant phenotype we observed in over 50 cells. We tested several commercially available antibodies reported to be against Hhat but none of these recognized overexpressed transfected C-terminally V5-tagged Hhat (data not shown). Attempts to raise our own antibodies to Hhat have so far been unsuccessful. We are therefore unable at present to confirm the intracellular localization of endogenous Hhat.

### siRNA Knockdown of Hhat in PANC1 and HEK-Hhat-V5 cells

**Figure 2:**
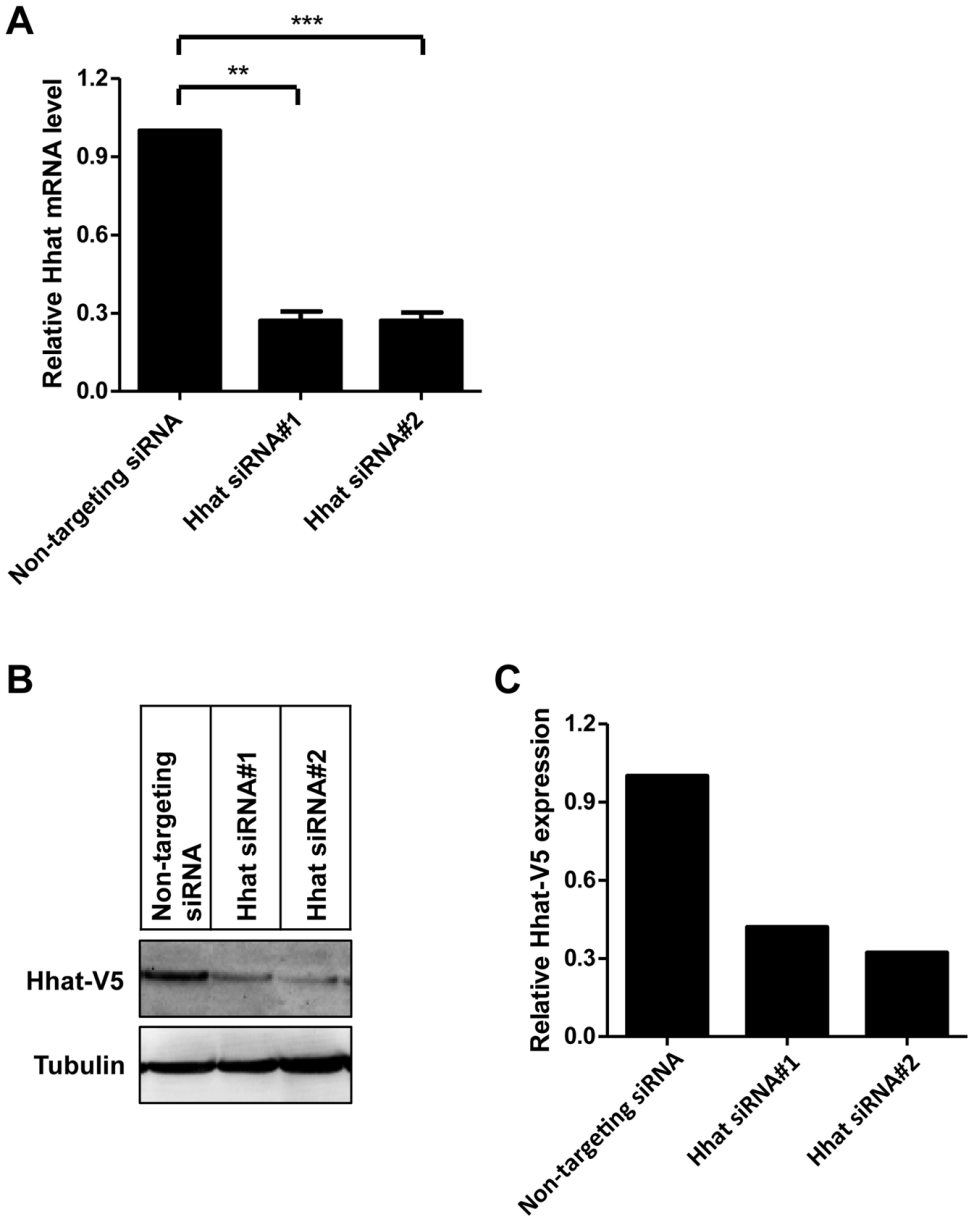
Hhat RNAi KD in PANC1 and HEK293a-Hhat-V5 cells. A. Quantitative RT-PCR was performed using Hhat-specific primers to confirm target gene knockdown in PANC1 cells following Hhat-#1 and Hhat-#2 siRNA transfection. Hhat expression is normalized with GAPDH and compared to Non-targeting siRNA control. Error bar represents the standard error of at least three independent experiments performed in duplicate (**, *P*<0.01; ***, *P*<0.001). B. To confirm Hhat KD at the protein level, HEK293a cells stably expressing a pcDNA-DEST40-Hhat-V5 construct at moderate levels were transfected with the Hhat-#1, Hhat-#2 and Non-targeting siRNAs. 72 h post transfection cells were lysed and Hhat expression examined by SDS-PAGE followed by western blotting with an anti-V5 antibody. Blots were probed for tubulin as a loading control. C. Densitometry was performed on the blots in panel B; values were then normalized to the tubulin loading control and compared to the Non-targeting siRNA control.

To determine whether Hhat is essential for Shh palmitoylation, we established conditions for effective KD of Hhat in PANC1 cells. The siRNA KD of Hhat was examined by qPCR using GAPDH as a loading control. In addition, to determine the specificity of Hhat siRNA, three negative controls were used: a non-targeting siRNA, a scrambled siRNA (Hhat-Scr) and a specific control mutated Hhat-#1 siRNA which contains mutations from the original Hhat-#1 siRNA in the 10^th^, 13^th^ and 19^th^ nucleotide to preclude siRNA activity. Our results show that optimized Hhat-#1 RNAi KD results in a >70% decrease in Hhat expression in PANC1 cells (Figure 2A), whereas with the non-targeting siRNA the Hhat level was unaffected (Figure 2A); similar results were observed with Hhat-Scr siRNA and Mutated Hhat-#1 siRNA treatment (data not shown). By qPCR, Hhat KD was quantified as 77% KD with Hhat-#1 and 68% KD with Hhat-#2 at 72 h compared to mutated Hhat-#1. Hhat KD achieved when data were compared to Hhat-Scr was 66% with Hhat-#1 and 71% KD with Hhat-#2 (data not shown). Similar KD of Hhat was measured by qPCR with HEK293a-Shh (Figure S2) and A549 cells (data not shown). Due to the lack of a specific Hhat antibody we were unable to directly confirm KD of endogenous Hhat at the protein level. However, to provide evidence that Hhat siRNA KD did effectively reduce Hhat protein levels, we stably expressed Hhat-V5 at moderate levels in HEK293a cells and then transfected the cells with Hhat siRNA-#1 or #2 for 72 h followed by anti-V5 immunoblotting. Figure 2B and C show that Hhat-#1 and #2 transfection substantially reduced Hhat-V5 expression by 58.1% and 67.8% respectively.

### Knocking down Hhat in PANC1 cells reduces endogenous Shh palmitoylation and oligomerization and decreases Shh cellular retention

**Figure 3:**
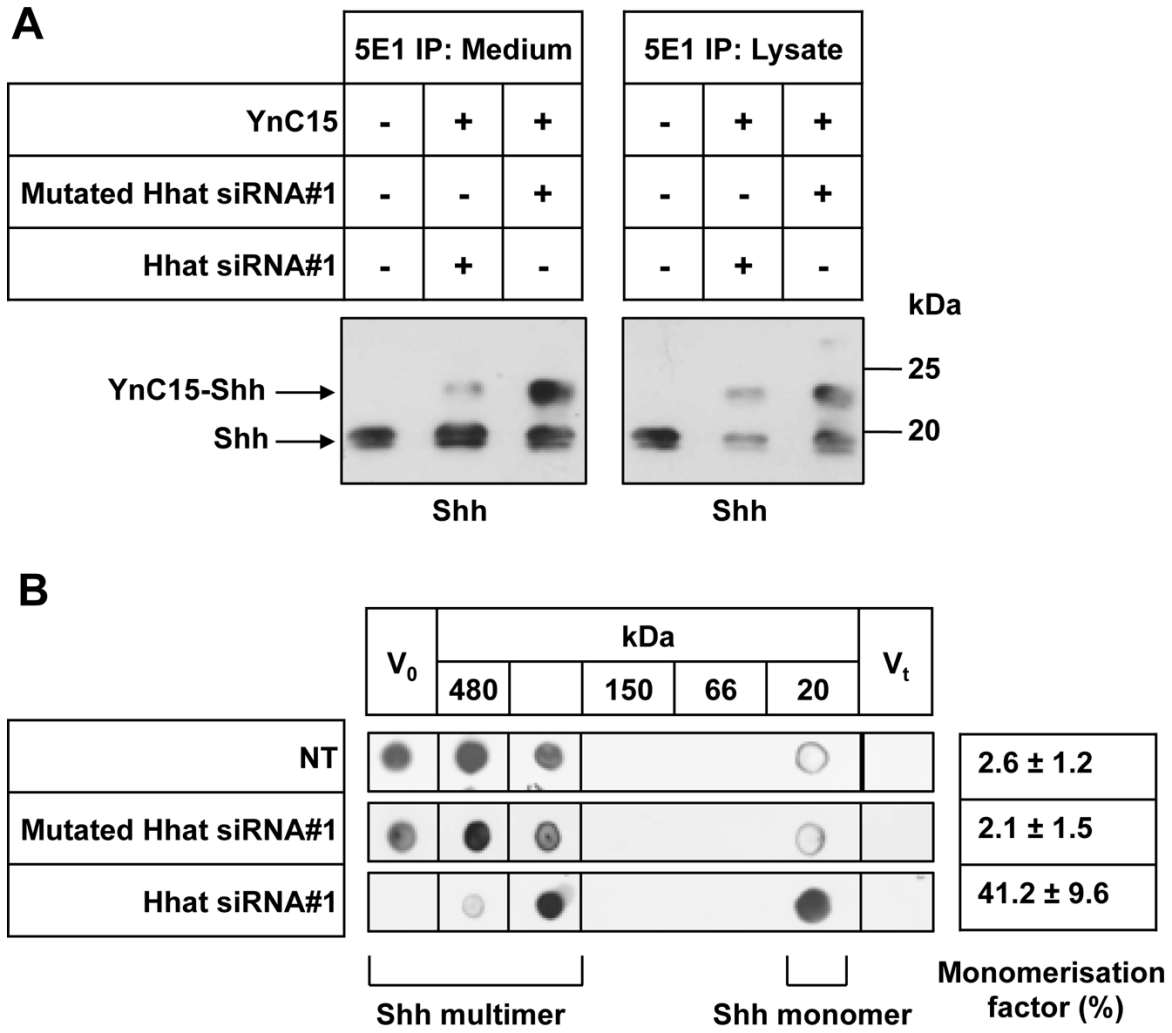
Hhat KD inhibits Shh palmitoylation and multimeric complex formation. A. 48 h after Hhat-#1 siRNA transfection PANC1 cells were labeled with YnC15, then medium and cell lysates were collected for 5E1 immunoprecipitation, treated by click chemistry and analyzed by Shh Western blot with H-160 Ab. In both medium and cell lysates, YnC15-labeled Shh was reduced in Hhat-#1 KD cells compared to Mutated Hhat-#1 and control cells. In contrast, YnC15-unlabeled Shh was more abundant in the medium of Hhat-#1 KD cells, but less abundant in cell lysates, demonstrating increased release when Hhat is knocked down. B. To examine the role of Hhat in Shh oligomerization, 72 h after siRNA transfection of PANC1 cells the media were subjected to gel filtration chromatography (Superdex200 10/300 GL column). After TCA precipitation, the fractions were probed by dot blot with anti-Shh H-160 antibody. Untreated and control Mutated Hhat-#1-treated cells showed an abundance of large complexes migrating near the void volume (Vo), whereas Hhat-#1 siRNA-treated KD cells had a much higher proportion of monomer (Vt represents the total volume of the column). Experiments were repeated three times with similar results.

We established a cell-based Shh palmitoylation assay using the bioorthogonal Cu(II)-catalyzed azido-alkyne cycloaddition reaction, also called click chemistry [29], [30]. In short, proteins metabolically incorporate the palmitate analogue YnC15 and are detected following reaction with an azido-TAMRA-biotin tag. Palmitoylation can be monitored by fluorescence imaging of the tag and also by anti-Shh immunoblotting which detects a slightly larger (∼3–5 kDa) Shh polypeptide due to tag addition (see Figure S3). Figure 3A shows that YnC15-tagged Shh could be immunoprecipitated by 5E1 MAb from cell lysate and medium of control PANC1 cells treated with Hhat-Mut siRNA. Hhat-#1 siRNA treatment, however, caused a substantial reduction in YnC15-labeled Shh in both lysate and medium, directly demonstrating inhibition of Shh palmitoylation by Hhat KD. Interestingly, in the medium of Hhat-#1 KD cells YnC15-unlabeled Shh was more abundant than that of control cells, but was less abundant in cell lysates, suggesting increased release when Hhat is knocked down. Similar results were obtained in HEK293a-Shh cells using Hhat-#1 and Hhat-#2 siRNAs (data not shown).

To evaluate the role of Hhat in formation of Shh multimeric complexes, gel filtration followed by Shh dot blotting were carried out on PANC1 conditioned medium. Using H-160 anti-Shh dot blotting (Figure 3B), we showed that most secreted Shh in control PANC1 culture medium migrated as large multimeric complexes and only a small portion of Shh migrated as monomers, similarly to previous studies [15], [31]. We analyzed whether Hhat was involved in this multimeric complex formation by Hhat siRNA treatment. In conditioned medium from Hhat-#1 siRNA-treated cells, a large proportion of Shh monomers was detected (41.2±9.6%), nearly 20 times higher than in control medium, whereas Mutated Hhat-#1 siRNA-treated medium shows a similar pattern to control medium with only 2.1±1.5% monomer. These data suggest that KD of Hhat in PANC1 cells causes decreased Shh multimer secretion and confirms that palmitoylation of Shh plays a positive role in Shh multimer formation [12].

### Knocking down Hhat causes decreased signaling through the Shh pathway in PANC1 cells

**Figure 4:**
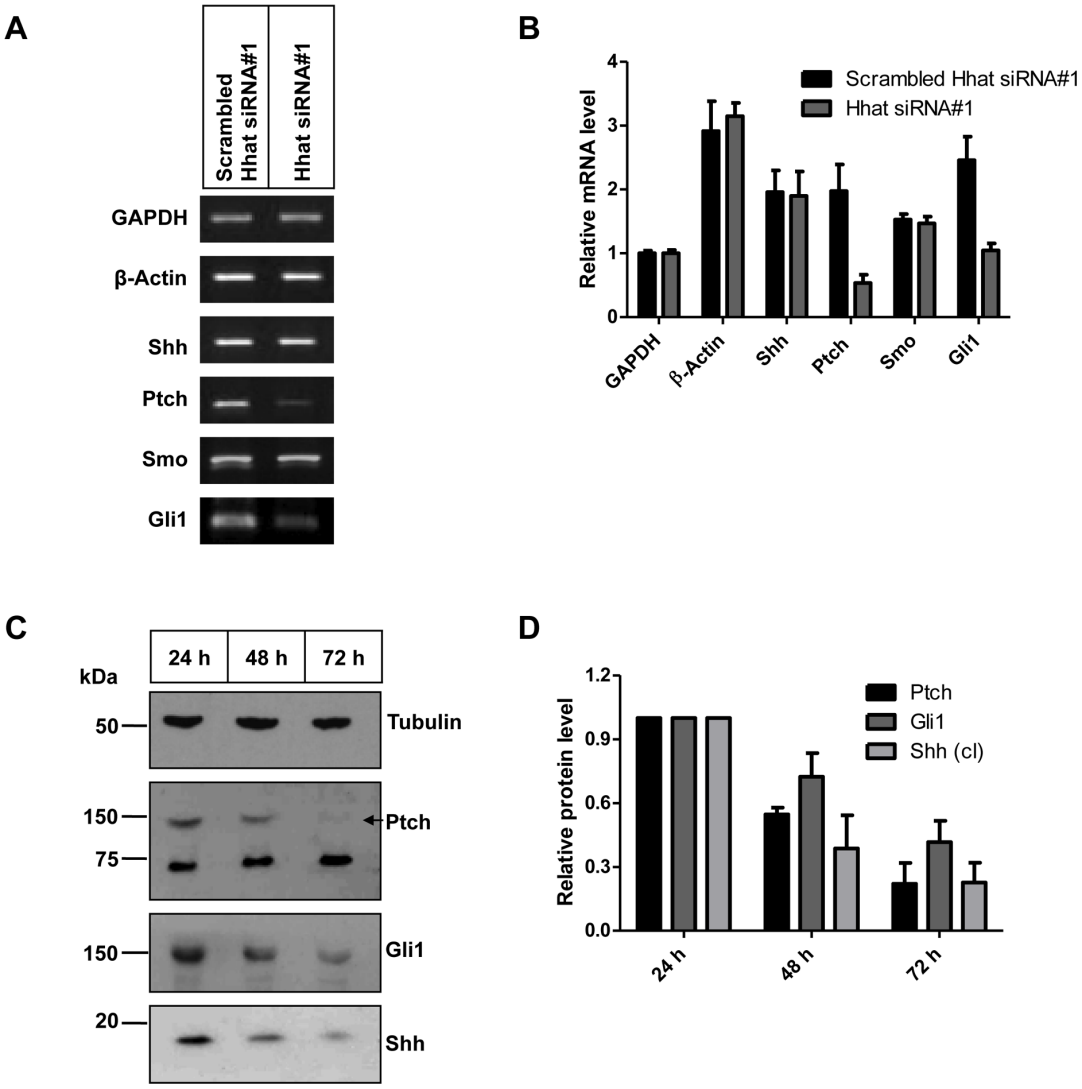
Hhat KD reduces Shh signaling in PANC1 cells. To study the role of Hhat in Shh signaling, expression levels of Shh targets Ptch and Gli1 were examined by both RT-PCR and Western blot after Hhat-#1 KD (A, C). Quantification of results is provided in B and D. Interestingly, cell-associated Shh protein is reduced by Hhat-#1 KD (C, bottom panel) but Shh mRNA level is unchanged (A), suggesting that Hhat-#1 KD reduces Shh retention by cells. In combination, this suggests that un-palmitoylated Shh has less ability to associate to cell membranes, but instead is released to the medium.

To determine whether Hhat (and hence palmitoylation of Shh) is required for Shh signaling activity, as indicated by Hhat knockout mice studies [12], RT-PCR and immunoblotting were performed in a Hhat-#1 KD time-course experiment; GAPDH and β-Actin were used as loading controls. After Hhat-#1 siRNA treatment, Ptch and Gli1 mRNA level were decreased in PANC1 cells by ∼70% and ∼50% respectively, whereas Shh and Smo showed no significant difference (Figure 4A, B). In PANC1 cells, Ptch and Gli1 were also similarly reduced at the protein level (Figure 4C, D). Interestingly, when we examined Shh by immunoblotting of cell lysates, cell-associated Shh was reduced ∼70% at 72 h after Hhat-#1 siRNA treatment (Figure 4C), consistent with non-palmitoylated Shh not being retained by cells. This reduction was not due to alteration in Shh mRNA level (Figure 4A, B). These data show that Hhat KD causes decreased signaling through the Hh pathway in PANC1 cells and reduced Shh association with the cells, suggesting that non-palmitoylated Shh is poorly retained at the cell surface. To confirm that the effects of Hhat siRNA knockdown in PANC1 cells are due to reduced canonical Hedgehog signalling we tested whether inhibition of Shh or Smo phenocopies the effect of Hhat siRNA KD. This is critical as PANC1 cells have been reported to be unresponsive to Smo inhibition, displaying Hh/Smo-independent Gli activation (e.g. by TGFβ, [51]). There are conflicting data in the literature highlighted by the differences seen in PANC1 cells responsiveness to the Hh pathway [8], [51] (and references in file S1). We therefore inhibited Smo with GDC0449 and Shh with the antagonist Robotnikin in PANC1 cells, using qPCR for Gli1 as a readout of Hh pathway activity. Figure S4 shows significant inhibition of Gli1 expression by both inhibitors, indicating that Smo and Shh are contributing to downstream Hh signaling in these cells. Differences in the literature may reflect the properties of different strains of PANC1 cells, or different experimental approaches.

### PANC1 proliferation is Shh-dependent and inhibited by knocking down Hhat

**Figure 5:**
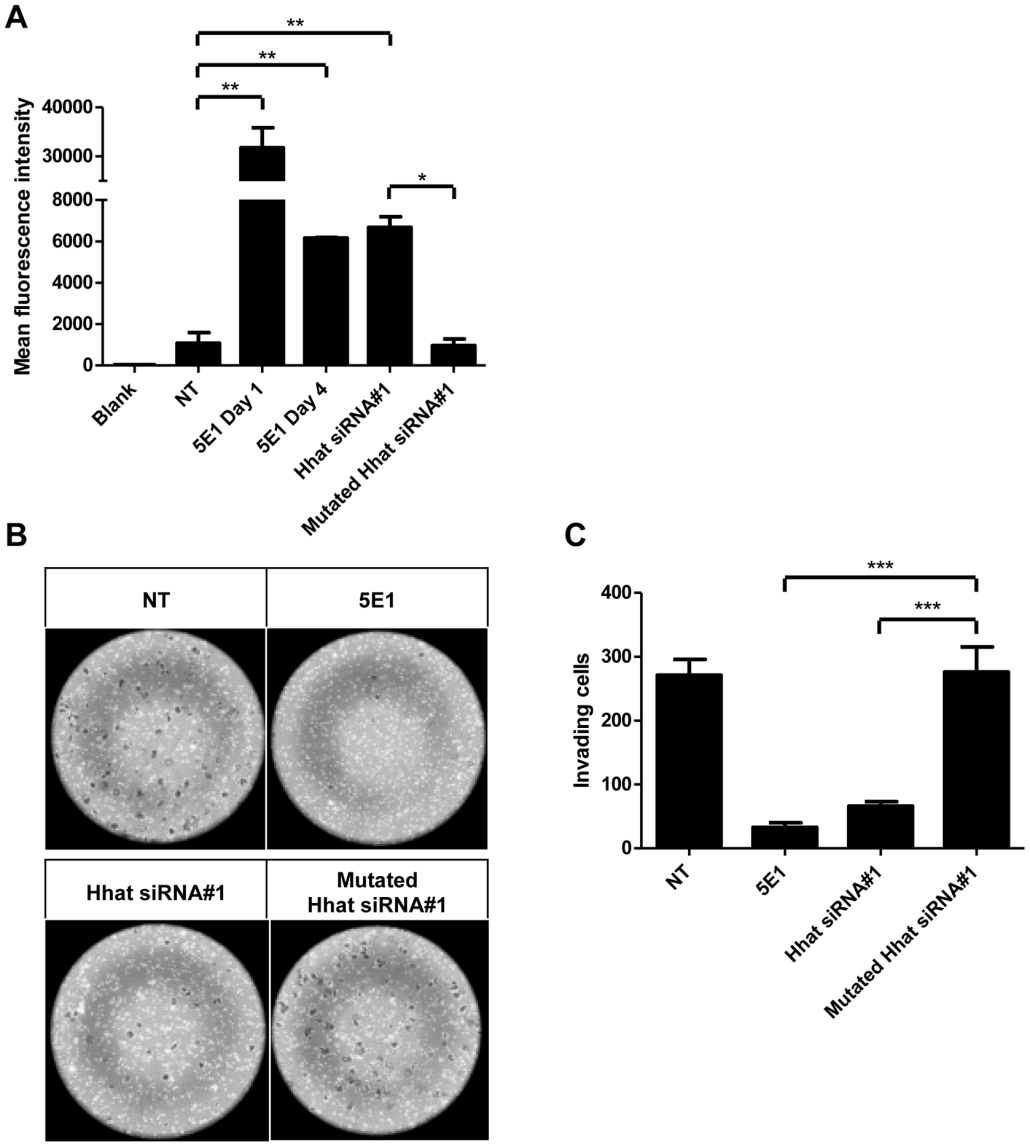
Hhat KD inhibits PANC1 proliferation and matrix invasion. A. PANC1 cells were labeled with CFSE at the start of the experiment and treated with Shh neutralizing antibody 5E1 on Day 1 (5E1 D1) to test whether PANC1 proliferation is Shh dependent. 5E1 treatment on Day 4 (5E1 D4) was used to mimic the kinetics of siRNA KD. Cells were allowed to grow for 8 days during which CFSE dilution gave a measure of cell division. 8 days after transfection with Hhat-#1 siRNA the cells were analyzed by flow cytometry. The Y-axis shows the CFSE mean fluorescence intensity (MFI) observed from 30,000 cells in each condition. The experiments were repeated three times in triplicate, and statistical significance was measured by using the two-tailed t test (***, *P*<0.001). B, C. 72 h after transfection with Hhat-1 siRNA or control Mutated Hhat siRNA PANC1 cells were plated onto Matrigel invasion chambers for 24 h. Cells that had migrated from the upper to the lower side of the filter were photographed (B) and counted with a light microscope (40 fields/filter, C). Non-treated (NT) and 5E1-treated cells are shown for comparison in B. The experiments were repeated four times, and statistical significance was measured by using the two-tailed t test (***, *P*<0.001).

To determine the effects of Hhat on PANC1 proliferation, Hhat-#1 siRNA knockdown combined with CFSE incorporation were used, followed by flow cytometry analysis. We investigated whether PANC1 proliferation is Shh-dependent by treating cells with 5E1 inhibitory antibody for Shh. CFSE intensity in cells treated with 5E1 on day 1 (5E1 D1 group) was ∼30 times higher than untreated cells (Figure 5A), suggesting that untreated PANC1 cells divide ∼4 times faster than 5E1-treated cells over 8 days. Moreover, cells treated with 5E1 on day 4 (5E1 D4 group; used to mimic the kinetics of Hhat-#1 siRNA KD) showed ∼8 times higher CFSE intensity compared to untreated cells (NT). Both results show that PANC1 proliferation was Shh-dependent.

Seven days after transfection, Hhat-#1 siRNA-treated cells had a significantly higher (∼8 times) CFSE fluorescence (>6000) compared to control Mutated Hhat-#1 siRNA-transfected cells (<1000) (Figure 5A). These results indicate that siRNA-mediated KD of Hhat suppresses PANC1 cell proliferation substantially.

We used a different cell proliferation assay to confirm these data in the A818-1 cell line, established from ascites of a 75 year old female with a differentiated PDAC [34]. These cells expressed significant amounts of Shh, as determined by Western blotting (data not shown). Hhat siRNA KD was optimized for this cell line and KD efficiency of 80% was achieved with Hhat siRNA #1 and 90% with Hhat siRNA #2 .as determined by qPCR (data not shown). 5×10^3^ A818-1 cells were transfected with Hhat-#1, Hhat-#2 or non-targeting siRNA in 96-well plates. Cell proliferation was monitored by measuring DNA content using the CyQUANT NF cell proliferation assay. Proliferation was significantly inhibited in the Hhat-#1 and Hhat-#2 siRNA-transfected cells compared to non-targeting siRNA control at 48 h post-transfection, and for Hhat-#1 siRNA at 96 h post-transfection (Figure S5). This result confirms that Hhat KD in PDAC cell lines significantly inhibits cell proliferation.

### Knocking down Hhat inhibits PANC1 invasion

To investigate the contribution of Hhat to the invasive potential of PANC1 cells, we carried out invasion assays after Hhat KD. We have previously reported that PANC1 invasion is dependent on Shh signaling using the blocking antibody 5E1 [15]. When PANC1 cells were transfected with Hhat-#1 siRNA 24 h prior to the invasion assay, we observed a significant decrease in the number of invasive PANC1 cells (66±7 cells/well), a level similar to that previously observed for 5E1 treatment (Figure 5B, C). In contrast, Mutated Hhat-#1 siRNA control treatment gave similar levels (276±40 cells/well) to untreated control cells [15]. These results indicate that expression of Hhat was required for efficient PANC1 invasion. Importantly, direct cell growth assays showed that the doubling time for PANC1 cells is ∼51 h (data not shown). The four-fold reduction in invasion observed in 24 h cannot therefore be solely accounted for by inhibition of cell growth.

### Hhat siRNA KD reduces juxtacrine/paracrine signaling

**Figure 6:**
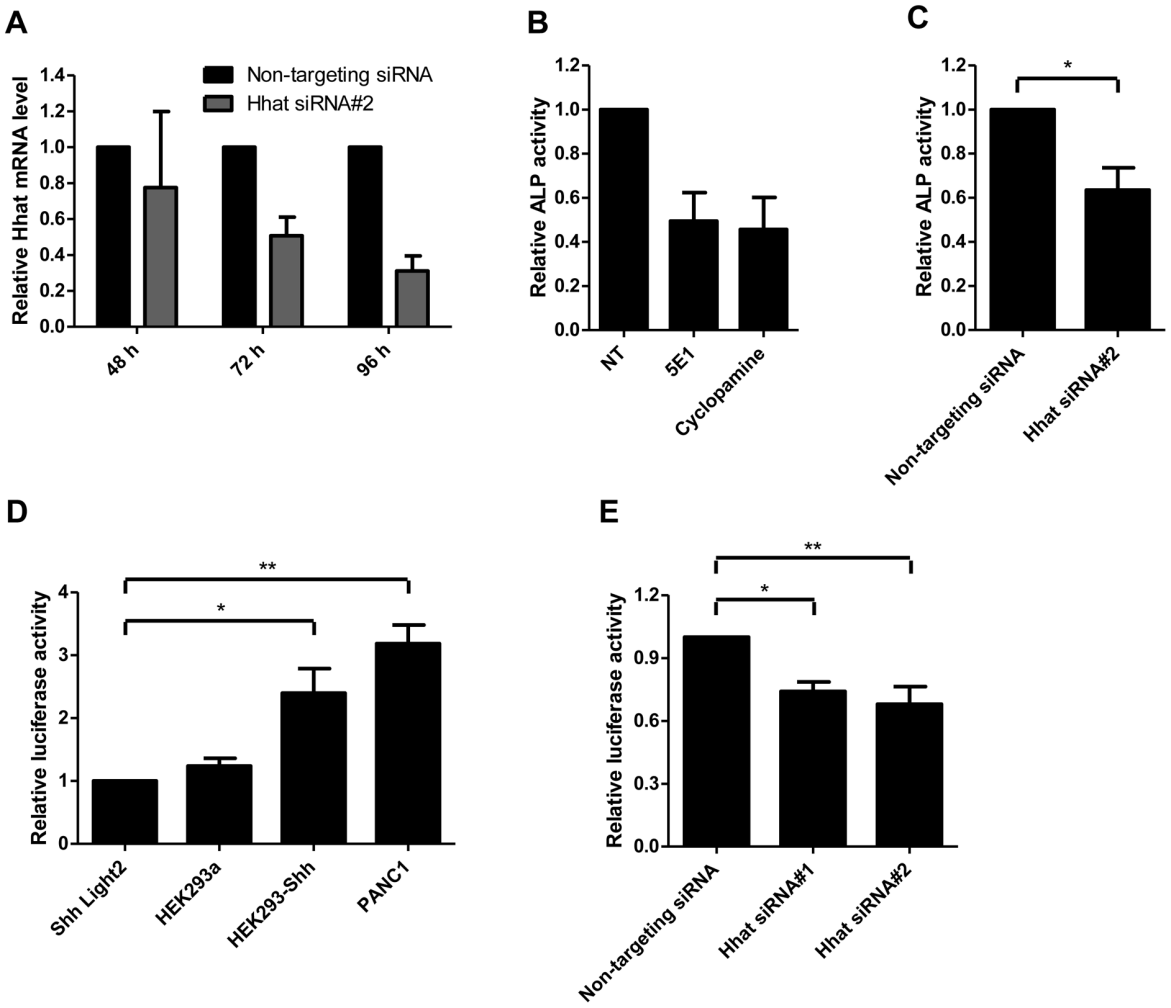
Hhat KD inhibits Hh-mediated juxtacrine/paracrine signaling. A. Hhat-#2 siRNA KD in A549 cells was assessed by qPCR 24–96 h after siRNA transfection showing 20–60% decrease in Hhat mRNA expression. B. A549 cells co-cultured with C3H10T1/2 cells (1∶2 ratio) were treated with cyclopamine or 5E1 anti-Shh blocking antibody for 2 days and ALP induction in C3H10T1/2 cells was then determined using p-nitrophenyl phosphate in an ALP assay and measuring absorbance at 405 nm. C. A549 cells treated with Hhat-#2 siRNA for 2 days were co-cultured with C3H10T1/2 cells (1∶2 ratio) and ALP induction was assessed after additional 2 days as in B. The results show a significant reduction (*, *P*<0.05) in the ability of A549 cells treated with Hhat-#2 compared to cells treated with non-targeting (NT) siRNA pool to induce ALP production in C3H10T1/2 cells. The experiments were repeated four times and statistical significance was measured using the paired t test. D. PANC1 and HEK293a-Shh cells induce luciferase activity in the Shh-Light2 cells when co-cultured for 72 h, while the HEK29a cell line, which does not express Shh, did not show luciferase activity higher than background levels (Shh-Light2 cells alone). The data shown are representative of five independent experiments and are the means ± standard error of triplicates. Data were normalized to the Shh-Light2 cell monoculture response (*, *P*<0.05; **, *P*<0.01). E. PANC1 cells were transfected with Hhat-#1, Hhat-#2 or Non-targeting siRNAs. 48 h post-transfection, Shh-Light2 cells were mixed with the transfected cells (2∶1 ratio) and they were co-cultured for 72 h. The data shown are representative of three independent experiments and are the means ± standard error of triplicates. Data were normalized to the Non-targeting siRNA response (*, *P*<0.05; **, *P*<0.01).

We established an assay for juxtacrine/paracrine Hh signaling using the reporter C3H10T1/2 cells, a mouse osteoblast progenitor line which responds to Shh stimulation by differentiating into osteoblasts [35], [36], which is marked by induction of alkaline phosphatase (ALP) after 2–5 days stimulation with Hh proteins. We tested A549 human NSCLC cells which reportedly express Shh [10], [37]; to test if Shh produced by A549 cells was able to induce a Hh response in surrounding cells in a juxtacrine/paracrine fashion, A549 cells were co-cultured with C3H10T1/2 cells. siRNA treatment of A549 cells resulted in 20–60% KD of Hhat mRNA when assayed by qRT-PCR normalized to GAPDH expression (Figure 6A). The induction of ALP in C3H10T1/2 cells by A549 cells was inhibited by Shh-blocking antibody 5E1 (20 µg/ml) and cyclopamine (5 µM) confirming that the ALP response in C3H10T1/2 cells was largely due to Shh produced by A549 cells (Figure 6B). After two days of Hhat-#2 siRNA treatment, A549 cells were co-cultured with C3H10T1/2 cells (ratio 1∶2 A549:C3H10T1/2) for two days. The C3H10T1/2 cell response by ALP production was reduced by 37±10% (*p* = 0.036) compared to treatment with a non-targeting pool of siRNAs (Figure 6C), showing that Hhat KD indeed leads to a reduction in juxtacrine/paracrine Shh signaling by A549 NSCLC cells.

To confirm that the response in C3H10T1/2 cells was due to Shh produced by co-cultured cells, a stable clone of HEK239a cells expressing plasmid pcDNA-DEST40-Shh, HEK293a-Shh, was created and was morphologically indistinguishable from HEK293a wt. Click chemistry experiments further confirmed that Shh was efficiently palmitoylated in these cells (Figure S3). HEK293a-Shh cells induced differentiation of C3H10T1/2 cells while HEK293a wt cells did not. Although HEK293a cells did not express enough endogenous Shh to be able to induce C3H10T1/2 differentiation, they expressed Hhat which palmitoylated exogenous Shh and which could be modulated by Hhat siRNA. Hhat siRNA treatment of HEK293a-Shh cells assayed by qPCR on day 2, 3 and 4 showed a decrease in Hhat mRNA (Figure S2 and data not shown). The level of intracellular Shh in HEK293a-Shh cells analyzed by Western blotting showed that Hhat-#2 KD resulted in a reduction of intracellular Shh, similar to that seen in PANC1 cells in Figure 3A and 4C, consistent with the role of Shh palmitoylation in the cell as a membrane anchor for the protein (data not shown).

HEK293a-Shh cells treated with Hhat-#2 siRNA co-cultured with C3H10T1/2 cells (ratio 1∶2) for 2 days during day 2–4 of KD, resulted in a C3H10T1/2 response decrease of 32±10% compared to non-targeting siRNA, but this did not quite reach statistical significance. We therefore used the well characterized Shh-Light2 cell line [38] as a reporter of juxtacrine/paracrine signaling from HEK293a-Shh cells. Shh-Light2 cells contain a luciferase gene under the control of a Gli-responsive promoter and respond to Shh stimulation by inducing Luciferase activity. Figure 6D shows that HEK293a-Shh cells but not control HEK293a cells were able to induce luciferase in co-culture with Shh-Light2 cells. PANC1 cells also induced luciferase expression by Shh-Light2 cells (Figure 6D), and Hhat KD by Hhat-#2 siRNA treatment reduced this by 44.1% (*p* = 0.0051) (Figure 6E).

## Discussion

The increased Hh signaling detected in many types of cancer and the identification of the significant role it plays in regulating the stromal environment to promote cancer growth and metastasis, has identified the Hh signaling pathway as a valid target in cancer. Smo inhibitors, currently under development by several companies, have great potential but mutations in Smo that make tumour cells resistant will make them ineffective in time, as has been found for many drug targets [39]. In this work, we present evidence that inhibiting the post-translational palmitoylation of Shh, by knocking down the enzyme Hhat responsible for palmitoylating Shh, inhibits PDAC cell growth and invasion and Shh signaling.

Hhat localization was confirmed to be primarily in the ER with little if any presence in the Golgi complex. This was done in two separate cell lines, using transfection with a Hhat-GFP fusion construct in human PANC1 cells, and a similar result was obtained for Hhat-V5 in transfected HEK293a cells. These data confirm the major site of Hhat localization as the ER, but in contrast to work published by Resh and colleagues in transfected COS-1 cells [33], [40] we found very little if any localization to the Golgi complex. These differences may be due to the use of different cell types. Using novel bioorthogonal ligation chemistry [28], [29] we have shown that Hhat KD caused near total ablation of Shh palmitoylation. Hhat KD also resulted in decreased cell-associated Shh, as expected if Shh acylation was inhibited. Inhibition of Shh palmitoylation also caused ablation of multimeric complex formation, the molecular species which is believed to be efficiently transported between Shh producing and receiving cells and to be biologically most active.

PANC1 cells have been reported to depend for their growth on Shh [8]. Using semi-quantitative RT-PCR and qPCR we show here that Hhat mRNA could be effectively knocked down in PANC1 cells by siRNA treatment for 48–72 h and that this resulted in reduction of target gene expression (Ptch, Gli). Off-target effects of siRNAs are of course possible but we have controlled for these by using control non-targeting, scrambled siRNA (Hhat-Scr) or a control siRNA mutated at every third base to abrogate interaction with Hhat mRNA which were ineffective, confirming the specificity of the effect. In addition, no off-target effects were observed on several other genes (Figure 4A, B). We used a proliferation assay based on the cytoplasmic fluorescent marker CFSE to monitor the effect of Hhat KD on PANC1 proliferation. Hhat-#1 siRNA (but not Mutated Hhat-#1) caused a strong reduction in cell division over 4 days, comparable to that seen with the Shh-neutralizing mouse monoclonal antibody 5E1. A Matrigel invasion assay was employed to show that the invasive properties of PANC1 cells were also dramatically curtailed by siRNA KD of Hhat (but not by control Mutated Hhat-#1 siRNA), similar to the effect of 5E1 antibody. Similar results were obtained in another PDAC cell line, A818-1. Moreover, the decrease in invasiveness over 24 h of PANC1 cells depleted of Hhat could not be accounted for by the reduction in cell proliferation during this time. This provides convincing evidence that Hhat is required for Shh signaling, proliferation and invasive behaviour of the PANC1 human PDAC cell line and that Hhat KD reverts the transformed properties of these cells. These results are supported by a recent paper [41] which showed that Hhat knockdown in NSCLC inhibited Shh palmitoylation as well as growth and survival of the tumor cells.

It could be argued that reduced cell viability of PANC1 cells, A549 or HEK-Shh may account for the reduction of Hh pathway activity in the signal receiving C3H10T1/2 or Shh-light2 cells, although the end effect is the same, i.e. oncogenic signaling is inhibited. We have not addressed the mechanism of the effect in this manuscript and that would be the subject of further studies. Also, it seems unlikely that cell death could be accounting for the observed effects in all three cell lines, especially in the HEK-Shh cells which are an artificial line and have no dependence on Hh signaling for their proliferation or survival.

Hhat is an attractive target for therapeutic intervention in the Hh signaling pathway because it appears to be solely responsible for acylation of Hhs, and its only known substrates in man are Hh proteins. Indeed, a mouse knockout of Hhat (called Skn in [12]) has a phenotype very similar to a Shh knockout resulting in developmental defects and neonatal lethality, indicating the essential role of Hhat in production of active Hh proteins. Blocking Shh palmitoylation via Hhat inhibition should be highly selective and would provide a complementary strategy that could be used singly or, more likely, in combination with drugs targeted at signal transduction in the receiving cell or stroma, such as Smo. Inhibiting an essential Shh modification at the same time as Smo may be advantageous since, blocking the pathway at two points might make resistance mutations much less likely. Attacking the Hh pathway is an attractive strategy because it can not only directly inhibit tumor cell growth but also interferes with paracrine signaling between the tumor cells and surrounding stroma [42] which contributes synergistically to pancreatic tumor progression [43]. These tumor-stroma interactions can cause desmoplasia, reducing blood flow to the tumor, thereby preventing access of conventional anti-cancer agents in PDAC [44]. Drugs against Hhat should be efficacious, as blocking Hh pathway signaling in adult animals, at least for short periods, could effectively cause regression of pancreatic and other digestive tract tumors but had no obvious deleterious effects on the recipients [8]. As this manuscript was about to be submitted, Resh and colleagues reported the identification of selective small molecule inhibitors of Hhat from a screen of over 63,000 commercially available compounds [40]. These compounds affect Shh signaling in model *in vitro* assays although effects on the growth of tumour cells were not reported. In addition, two selective small molecule inhibitors of the Porc MBOAT member have been described [45], [46] which inhibit Wnt acylation and do not affect Hhat, and inhibitors of GOAT have also been reported [47], [48]. The Porc inhibitor C59 inhibited the growth of a Wnt-driven breast cancer cell line and Wnt signaling, both *in vitro* and in a mouse xenograft model, with no apparent toxicity in the mice [46]. This provides considerable promise that selective Hhat inhibitors could be identified and serve as possible therapeutics.

In order to find highly specific and selective pharmacological agents targeted at Hhat it is important to thoroughly characterize the biochemistry and cell biology of this acyltransferase. To date there is little cell biological or biochemical analysis of Hhat from any species. Buglino and Resh [33] reported some important characterization of Hhat enzymatic properties in detergent solution including mutational studies which confirmed roles for the conserved histidine 379 and the adjacent tryptophan 378 residues in catalytic function, albeit not an absolute requirement [49]. Future progress will require a thorough molecular characterization of Hhat in its native membrane environment, including determining its transmembrane topology and crucial catalytic residues by mutational analysis.

## Supporting Information

Figure S1 **Transmembrane topology of Hhat predicted using the TOPCONS Programme.**
(TIF)

Figure S2 **Hhat mRNA is effectively knocked down by siRNA treatment in HEK293a-Shh cells.** Quantitative RT-PCR was performed using Hhat-specific primers to confirm target gene knockdown in HEK293a-Shh cells following Hhat-#1 and Hhat-#2 siRNA transfection. Hhat expression is normalized to GAPDH and compared to Non-targeting siRNA control. Error bar represents the standard error of three independent experiments performed in triplicate (*, P<0.05).
(TIF)

Figure S3 **HEK-Shh stable cell line produces palmitoylated Shh.** HEK-Shh cells were grown overnight in media that contained YnC15 or palmitic acid. Cell lysates were prepared and then treated by click chemistry. Labeled proteins were pulled down on Neutravidin-coated beads and pulled down proteins were analyzed by SDS-PAGE and in gel fluorescence (A) and subsequently by Western blotting using Shh and tubulin antibodies (B). A. In-gel fluorescence of YnC15-labelled proteins from the HEK-Shh cell line (right hand 3 lanes). Control cells fed with palmitic acid (left hand 3 lanes) do not show any fluorescently-labeled proteins either in the input or following pull down with Neutravidin-coated beads, clearly indicating that fluorescence is due to the clicked proteins. B. Gel in image A was transferred to PVDF membrane and probed with anti-Shh Ab H-160. In the palmitic acid-fed cells, a single Shh band is seen at 20 kDa; however, in the YnC15-fed cells, two Shh bands are present, one at about 25 kDa, representing the YnC15-labeled tagged Shh, and another band at 20 kDa, representing the unlabeled Shh molecules. The increased molecular weight is due to the size of the azido-TAMRA-biotin capture reagent. This is further shown following Neutravidin pull down of the clicked proteins, as only the upper YnC15-labeled Shh is seen in the bound proteins lane, while in the lane containing the unbound fraction the lower band only is present.
(TIF)

Figure S4 **Smo and Shh inhibition by small molecule inhibitors in PANC1 cells reduces Shh signaling.** PANC1 cells were treated with either the Smo inhibitor GDC0449 (A) or the Shh-binding antagonist Robotnikinin (B) or DMSO control. Shh signaling was examined by qPCR using Gli1-specific primers 48 h after treatment. A. GDC0449 treatment significantly reduced Gli1 expression (p = 0.0279). B. Robotnikinin also reduced Gli expression but did not reach significance. These data indicate that autocrine Shh signaling can be inhibited in the PANC1 cells used in this study. Our data are supported by several published studies on PANC1 cells [1]–[3]. Gli1 expression is normalized with GAPDH and compared to DMSO control. Error bar represents the standard error of n = 4 (GDC0449 data) and n = 3 (Robotnikinin data) independent experiments performed in duplicate; data were analysed in Graphpad Prism by a two-tailed unpaired student t-test (**, P<0.05; *, P<0.1).
(TIF)

Figure S5 **Hhat KD inhibits A818-1 cell proliferation.** For each condition, 2×104 A818-1 cells were transfected with the indicated siRNA and then 5×103 cells were plated out in four 96-well plates. Proliferation was subsequently monitored at specific time points by measuring the DNA content using the CyQUANT NF reagent. The Y-axis shows the fluorescence measurements reported as arbitrary units (AU) in each well for the indicated condition. Measurements were made using an Ettan DIGE imager with excitation at 485 nm and emission detection at 530 nm. The experiments were repeated four times, and statistical significance was measured by using the two-tailed t test (*, P<0.05).
(TIF)

File S1 (DOCX)

## References

[1] Varjosalo M, Taipale J (2008) Hedgehog: functions and mechanisms. Genes Dev 22: 2454–2472. 18794343 10.1101/gad.1693608

[2] Adolphe C, Narang M, Ellis T, Wicking C, Kaur P, et al (2004) An in vivo comparative study of sonic, desert and Indian hedgehog reveals that hedgehog pathway activity regulates epidermal stem cell homeostasis. Development 131: 5009–5019. 15371305 10.1242/dev.01367

[3] di Magliano PM, Hebrok M (2003) Hedgehog signaling in cancer formation and maintenance. Nat Rev Cancer 3: 903–911. 14737121 10.1038/nrc1229

[4] Katano M (2005) Hedgehog signaling pathway as a therapeutic target in breast cancer. Cancer Lett 227: 99–104. 16112412 10.1016/j.canlet.2004.11.030

[5] Hatsell S, Frost AR (2007) Hedgehog signaling in mammary gland development and breast cancer. J Mamm Gland Biol Neoplasia 12: 163–173. 10.1007/s10911-007-9048-2 17623270

[6] Jacob L, Lum L (2007) Deconstructing the hedgehog pathway in development and disease. Science 318: 66–68. 17916724 10.1126/science.1147314 PMC3791603

[7] Thayer SP, di Magliano MP, Heiser PW, Nielsen CM, Roberts DJ, et al (2003) Hedgehog is an early and late mediator of pancreatic cancer tumorigenesis. Nature 425: 851–856. 14520413 10.1038/nature02009 PMC3688051

[8] Berman DM, Karhadkar SS, Maitra A, de Oca RM, Gerstenblith MR, et al (2003) Widespread requirement for Hedgehog ligand stimulation in growth of digestive tract tumors. Nature 425: 846–851. 14520411 10.1038/nature01972

[9] Watkins DN, Berman DM, Burkholder SG, Wang B, Beachy PA, et al (2003) Hedgehog signaling within airway epithelial progenitors and in small-cell lung cancer. Nature 422: 313–317. 12629553 10.1038/nature01493

[10] Yuan Z, Goetz JA, Singh S, Ogden SK, Petty WJ, et al (2007) Frequent requirement of hedgehog signaling in non-small cell lung carcinoma. Oncogene 26: 1046–1055. 16909105 10.1038/sj.onc.1209860

[11] Mann RK, Beachy PA (2004) Novel lipid modifications of secreted protein signals. Annu Rev Biochem 73: 891–923. 15189162 10.1146/annurev.biochem.73.011303.073933

[12] Chen MH, Li YJ, Kawakami T, Xu SM, Chuang PT (2004) Palmitoylation is required for the production of a soluble multimeric Hedgehog protein complex and long-range signaling in vertebrates. Genes Dev 18: 641–659. 15075292 10.1101/gad.1185804 PMC387240

[13] Kohtz JD, Lee HY, Gaiano N, Segal J, Ng E, et al (2001) N-terminal fatty-acylation of sonic hedgehog enhances the induction of rodent ventral forebrain neurons. Development 128: 2351–2363. 11493554 10.1242/dev.128.12.2351

[14] Callejo A, Torroja C, Quijada L, Guerrero I (2006) Hedgehog lipid modifications are required for Hedgehog stabilization in the extracellular matrix. Development 133: 471–483. 16396909 10.1242/dev.02217

[15] Chang SC, Mulloy B, Magee AI, Couchman JR (2011) Two distinct sites in sonic hedgehog combine for heparan sulfate interactions and cell signaling functions. J Biol Chem 286: 44391–44402. 22049079 10.1074/jbc.M111.285361 PMC3247953

[16] Farzan SF, Singh S, Schilling NS, Robbins DJ (2008) The adventures of sonic hedgehog in development and repair. III. Hedgehog processing and biological activity. Am J Physiol Gastrointest Liver Physiol 294: G844–849. 18239057 10.1152/ajpgi.00564.2007 PMC2694571

[17] Ohlig S, Pickhinke U, Sirko S, Bandari S, Hoffmann D, et al (2012) An emerging role of Sonic hedgehog shedding as a modulator of heparan sulfate interactions. J Biol Chem 287: 43708–43719. 23118222 10.1074/jbc.M112.356667 PMC3527956

[18] Ohlig S, Farshi P, Pickhinke U, van den Boom J, Hoing S, et al (2011) Sonic Hedgehog Shedding Results in Functional Activation of the Solubilized Protein. Dev Cell 20: 764–774. 21664575 10.1016/j.devcel.2011.05.010

[19] van den Brink GR (2007) Hedgehog signaling in development and homeostasis of the gastrointestinal tract. Physiol Rev 87: 1343–1375. 17928586 10.1152/physrev.00054.2006

[20] Lee JD, Treisman JE (2001) Sightless has homology to transmembrane acyltransferases and is required to generate active Hedgehog protein. Curr Biol 11: 1147–1152. 11509241 10.1016/s0960-9822(01)00323-2

[21] Micchelli CA, The I, Selva E, Mogila V, Perrimon N (2002) Rasp, a putative transmembrane acyltransferase, is required for Hedgehog signaling. Development 129: 843–851. 11861468 10.1242/dev.129.4.843

[22] Chamoun Z, Mann RK, Nellen D, von Kessler DP, Bellotto M, et al (2001) Skinny hedgehog, an acyltransferase required for palmitoylation and activity of the hedgehog signal. Science 293: 2080–2084. 11486055 10.1126/science.1064437

[23] Chang SC, Magee AI (2009) Acyltransferases for secreted signaling proteins. Mol Membr Biol 26: 104–113. 19169935 10.1080/09687680802706432

[24] Shindou H, Shimizu T (2009) Acyl-CoA:lysophospholipid acyltransferases. J Biol Chem 284: 1–5. 18718904 10.1074/jbc.R800046200

[25] Zhai L, Chaturvedi D, Cumberledge S (2004) Drosophila wnt-1 undergoes a hydrophobic modification and is targeted to lipid rafts, a process that requires porcupine. J Biol Chem 279: 33220–33227. 15166250 10.1074/jbc.M403407200

[26] Gutierrez JA, Solenberg PJ, Perkins DR, Willency JA, Knierman MD, et al (2008) Ghrelin octanoylation mediated by an orphan lipid transferase. Proc Natl Acad Sci USA 105: 6320–6325. 18443287 10.1073/pnas.0800708105 PMC2359796

[27] Turner TT, Bomgardner D, Jacobs JP (2004) Sonic hedgehog pathway genes are expressed and transcribed in the adult mouse epididymis. J Androl 25: 514–522. 15223840 10.1002/j.1939-4640.2004.tb02822.x

[28] Heal WP, Wickramasinghe SR, Leatherbarrow RJ, Tate EW (2008) N-Myristoyl transferase-mediated protein labelling in vivo. Org Biomol Chem 6: 2308–2315. 18563263 10.1039/b803258k

[29] Heal WP, Jovanovic B, Bessin S, Wright MH, Magee AI, et al (2011) Bioorthogonal chemical tagging of protein cholesterylation in living cells. Chem Commun 47: 4081–4083. 10.1039/c0cc04710d 21221452

[30] Fokin VV (2007) Click Imaging of Biochemical Processes in Living Systems. ACS Chem Biol 2: 775–778. 18154263 10.1021/cb700254v

[31] Dierker T, Dreier R, Petersen A, Bordych C, Grobe K (2009) Heparan Sulfate-modulated, Metalloprotease-mediated Sonic Hedgehog Release from Producing Cells. J Biol Chem 284: 8013–8022. 19176481 10.1074/jbc.M806838200 PMC2658095

[32] Nakajima A, Tomimoto A, Fujita K, Sugiyama M, Takahashi H, et al (2008) H. Inhibition of peroxisome proliferator-activated receptor gamma activity suppresses pancreatic cancer cell motility. Cancer Sci 99: 1892–1900. 19016747 10.1111/j.1349-7006.2008.00904.x PMC11160097

[33] Buglino JA, Resh MD, et al (2008) Hhat Is a Palmitoylacyltransferase with Specificity for N-Palmitoylation of Sonic Hedgehog. J Biol Chem 283: 22076–22088. 18534984 10.1074/jbc.M803901200 PMC2494920

[34] Lehnert L, Trost H, Schmiegel W, Röder C, Kalthoff H (1999) Hollow-spheres: a new model for analyses of differentiation of pancreatic duct epithelial cells. Ann NY Acad Sci 880: 83–93. 10415853 10.1111/j.1749-6632.1999.tb09512.x

[35] Nakamura T, Aikawa T, Iwamoto-Enomoto M, Iwamoto M, Higuchi Y, et al (1997) T. Induction of osteogenic differentiation by Hedgehog proteins. Biochem Biophys Res Commun (1997) 237: 465–469. 10.1006/bbrc.1997.7156 9268735

[36] Pathi S, Pagan-Westphal S, Baker DP, Garber EA, Rayhorn P, et al (2001) Comparative biological responses to human Sonic, Indian, and Desert hedgehog. Mech Dev 106: 107–117. 11472839 10.1016/s0925-4773(01)00427-0

[37] Chi S, Huang S, Li C, Zhang X, He N, et al (2006) Activation of the hedgehog pathway in a subset of lung cancers. Cancer Lett 244: 53–60. 16446029 10.1016/j.canlet.2005.11.036

[38] Taipale J, Chen JK, Cooper MK, Wang B, Mann RK, et al (2000) Effects of oncogenic mutations in Smoothened and Patched can be reversed by cyclopamine. Nature 406: 1005–1009. 10984056 10.1038/35023008

[39] Yauch RL, Dijkgraaf GJ, Alicke B, Januario T, Ahn CP, et al (2009) Smoothened mutation confers resistance to a Hedgehog pathway inhibitor in medulloblastoma. Science 326: 572–574. 19726788 10.1126/science.1179386 PMC5310713

[40] Petrova E, Rios-Esteves J, Ouerfelli O, Glickman JF, Resh MD (2013) Inhibitors of Hedgehog acyltransferase block Sonic Hedgehog signaling. Nat Chem Biol 9: 247–249. 23416332 10.1038/nchembio.1184 PMC3604071

[41] Rodriguez-Blanco J, Schilling NS, Tokhunts R, Giambelli C, Long J, et al (2012) The Hedgehog processing pathway is required for NSCLC growth and survival. Oncogene 32: 2335–2345. 22733134 10.1038/onc.2012.243 PMC3821972

[42] Yauch RL, Gould SE, Scales SJ, Tang T, Tian H, et al (2008) A paracrine requirement for hedgehog signaling in cancer. Nature 455: 406–410. 18754008 10.1038/nature07275

[43] Tian H, Callahan CA, DuPree KJ, Darbonne WC, Ahn CP, et al (2009) Hedgehog signaling is restricted to the stromal compartment during pancreatic carcinogenesis. Proc Natl Acad Sci USA 106: 4254–4259. 19246386 10.1073/pnas.0813203106 PMC2647977

[44] Olive KP, Jacobetz MA, Davidson CJ, Gopinathan A, McIntyre D, et al (2009) Inhibition of Hedgehog signaling enhances delivery of chemotherapy in a mouse model of pancreatic cancer. Science 324: 1457–1461. 19460966 10.1126/science.1171362 PMC2998180

[45] Chen B, Dodge ME, Tang W, Lu J, Ma Z, et al (2009) Small molecule-mediated disruption of Wnt-dependent signaling in tissue regeneration and cancer. Nat Chem Biol 5: 100–107. 19125156 10.1038/nchembio.137 PMC2628455

[46] Proffitt KD, Madan B, Ke Z, Pendharkar V, Ding L, et al (2013) Pharmacological inhibition of the Wnt acyltransferase PORCN prevents growth of WNT-driven mammary cancer. Cancer Res 73: 502–507. 23188502 10.1158/0008-5472.CAN-12-2258

[47] Barnett BP, Hwang Y, Taylor MS, Kirchner H, Pfluger PT, et al (2010) Glucose and Weight Control in Mice with a Designed Ghrelin O-Acyltransferase Inhibitor. Science 330: 1689–1692. 21097901 10.1126/science.1196154 PMC3068526

[48] Yang J, Zhao TJ, Goldstein JL, Brown MS (2008) Inhibition of ghrelin O-acyltransferase (GOAT) by octanoylated pentapeptides. Proc Natl Acad Sci USA 105: 10750–10755. 18669668 10.1073/pnas.0805353105 PMC2504781

[49] Buglino JA, Resh MD (2010) Identification of Conserved Regions and Residues within Hedgehog Acyltransferase Critical for Palmitoylation of Sonic Hedgehog. PLoS One 5: e11195. 20585641 10.1371/journal.pone.0011195 PMC2890405

[50] Sirivatanauksorn V, Sirivatanauksorn Y, Gorman PA, Davidson JM, Sheer D, et al (2001) Non-random chromosomal rearrangements in pancreatic cancer cell lines identified by spectral karyotyping. Int J Cancer 91: 350–358. 11169959 10.1002/1097-0215(200002)9999:9999<::aid-ijc1049>3.3.co;2-3

[51] Nolan-Stevaux O, Lau J, Truitt ML, Chu GC, Hebrok M, et al (2009) GLI1 is regulated through Smoothened independent mechanisms in neoplastic pancreatic ducts and mediates PDAC cell survival and transformation. Genes Dev 23: 24–36. 19136624 10.1101/gad.1753809 PMC2632164

