# Supplementary figures and images for: Attenuation of Hedgehog Acyltransferase-Catalyzed Sonic Hedgehog Palmitoylation Causes Reduced Signaling, Proliferation and Invasiveness of Human Carcinoma Cells
Source: PLoS One. 2014 Mar 7;9(3):e89899. doi: 10.1371/journal.pone.0089899 (PMC3946499; doi:10.1371/journal.pone.0089899)

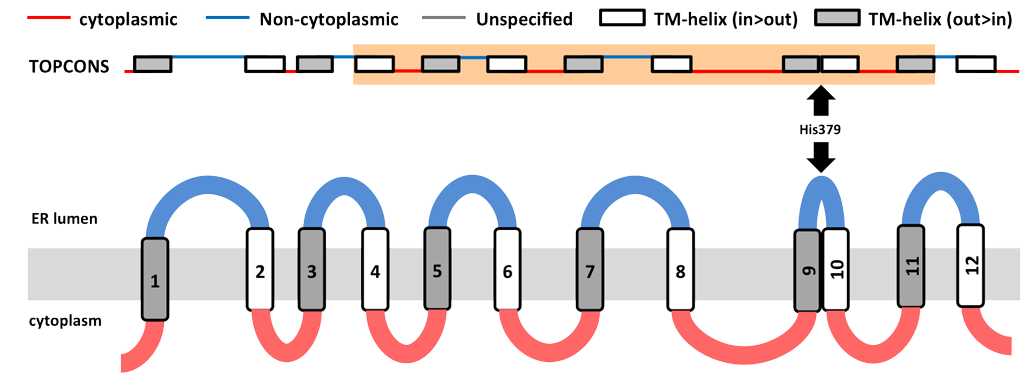

Supplement: Figure S1 — Transmembrane topology of Hhat predicted using the TOPCONS Programme. (TIF) [file pone.0089899.s001.tif]

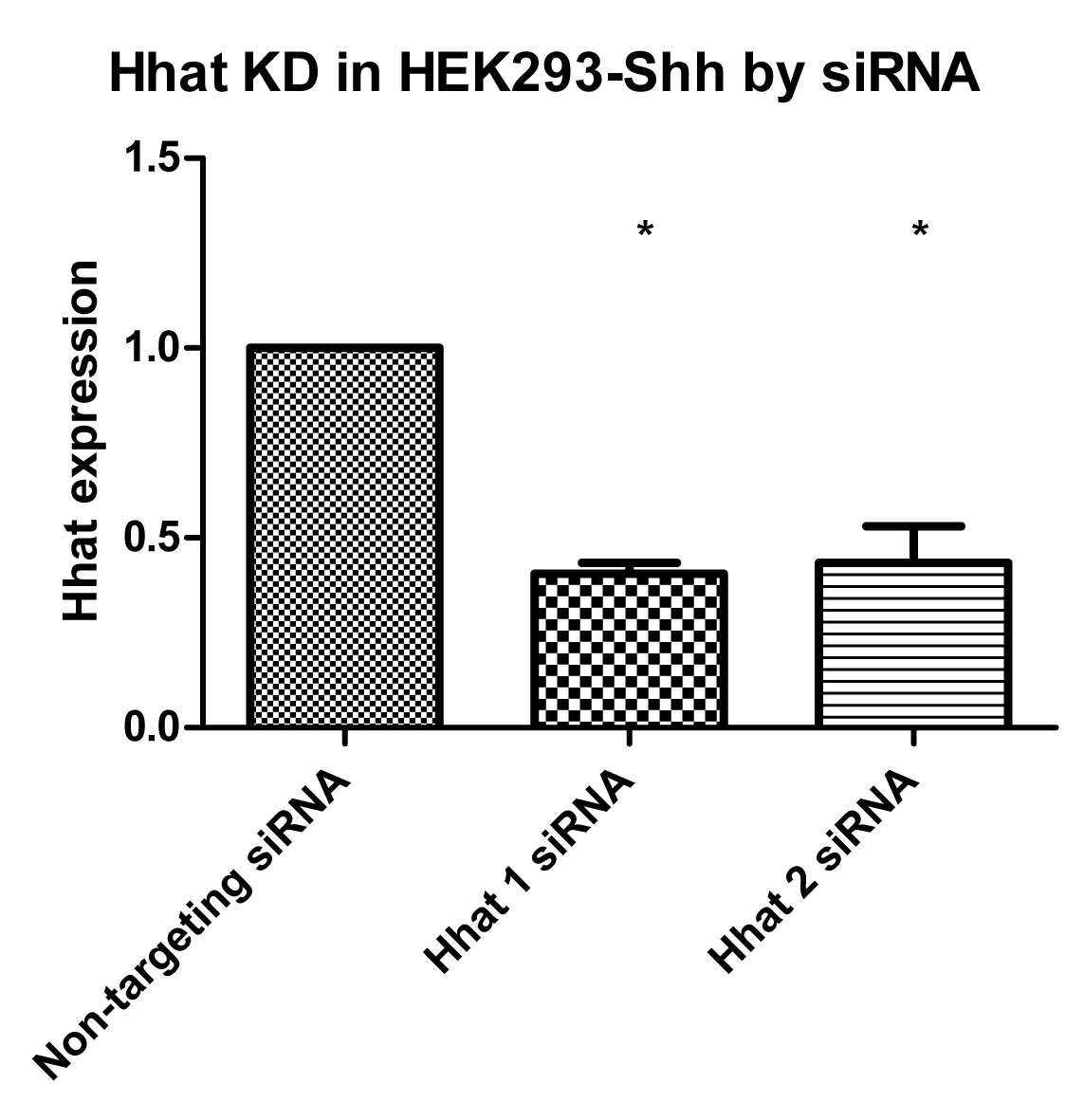

Supplement: Figure S2 — Hhat mRNA is effectively knocked down by siRNA treatment in HEK293a-Shh cells. Quantitative RT-PCR was performed using Hhat-specific primers to confirm target gene knockdown in HEK293a-Shh cells following Hhat-#1 and Hhat-#2 siRNA transfection. Hhat expression is normalized to GAPDH and compared to Non-targeting siRNA control. Error bar represents the standard error of three independent experiments performed in triplicate (*, P<0.05). (TIF) [file pone.0089899.s002.tif]

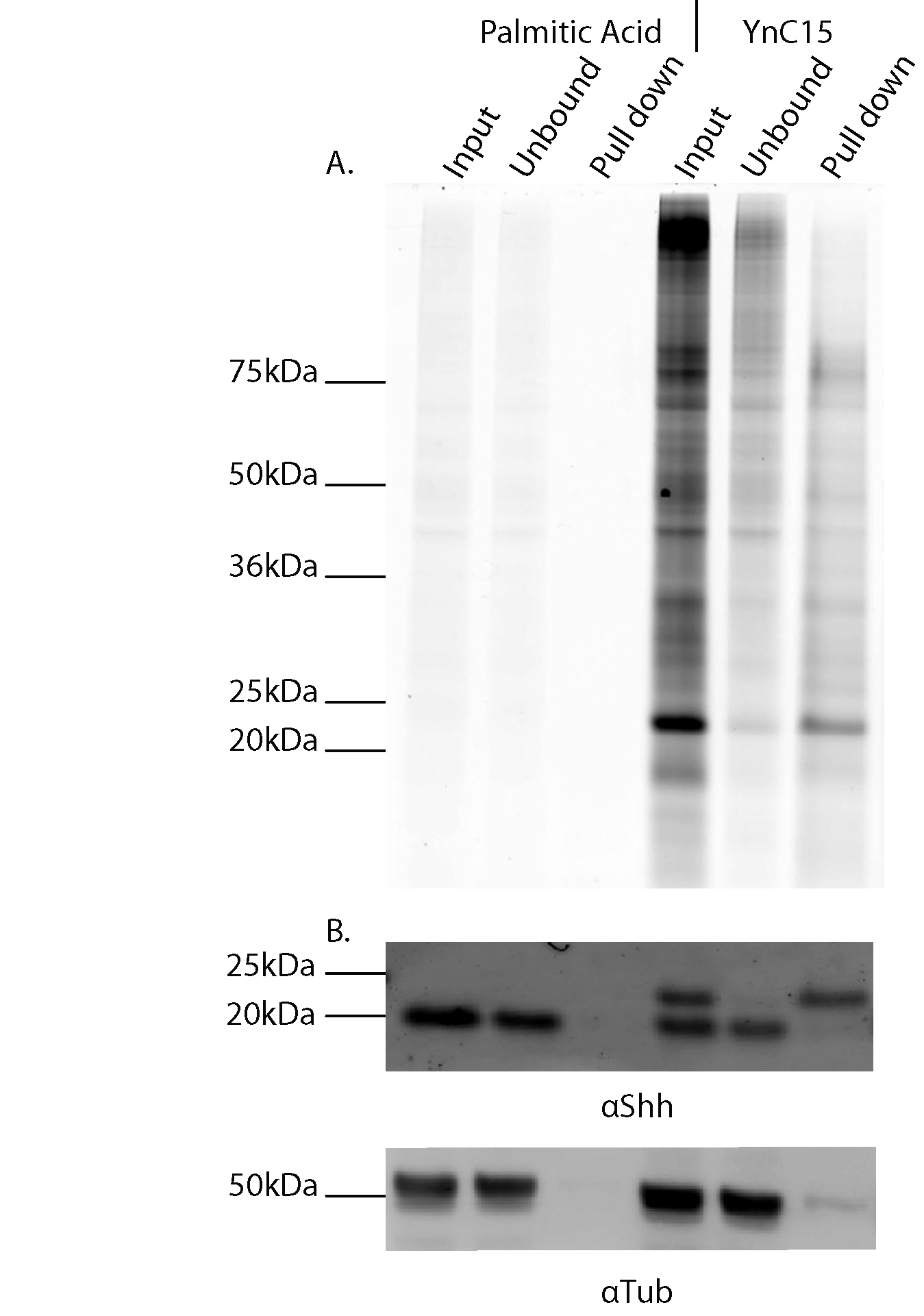

Supplement: Figure S3 — HEK-Shh stable cell line produces palmitoylated Shh. HEK-Shh cells were grown overnight in media that contained YnC15 or palmitic acid. Cell lysates were prepared and then treated by click chemistry. Labeled proteins were pulled down on Neutravidin-coated beads and pulled down proteins were analyzed by SDS-PAGE and in gel fluorescence (A) and subsequently by Western blotting using Shh and tubulin antibodies (B). A. In-gel fluorescence of YnC15-labelled proteins from the HEK-Shh cell line (right hand 3 lanes). Control cells fed with palmitic acid (left hand 3 lanes) do not show any fluorescently-labeled proteins either in the input or following pull down with Neutravidin-coated beads, clearly indicating that fluorescence is due to the clicked proteins. B. Gel in image A was transferred to PVDF membrane and probed with anti-Shh Ab H-160. In the palmitic acid-fed cells, a single Shh band is seen at 20 kDa; however, in the YnC15-fed cells, two Shh bands are present, one at about 25 kDa, representing the YnC15-labeled tagged Shh, and another band at 20 kDa, representing the unlabeled Shh molecules. The increased molecular weight is due to the size of the azido-TAMRA-biotin capture reagent. This is further shown following Neutravidin pull down of the clicked proteins, as only the upper YnC15-labeled Shh is seen in the bound proteins lane, while in the lane containing the unbound fraction the lower band only is present. (TIF) [file pone.0089899.s003.tif]

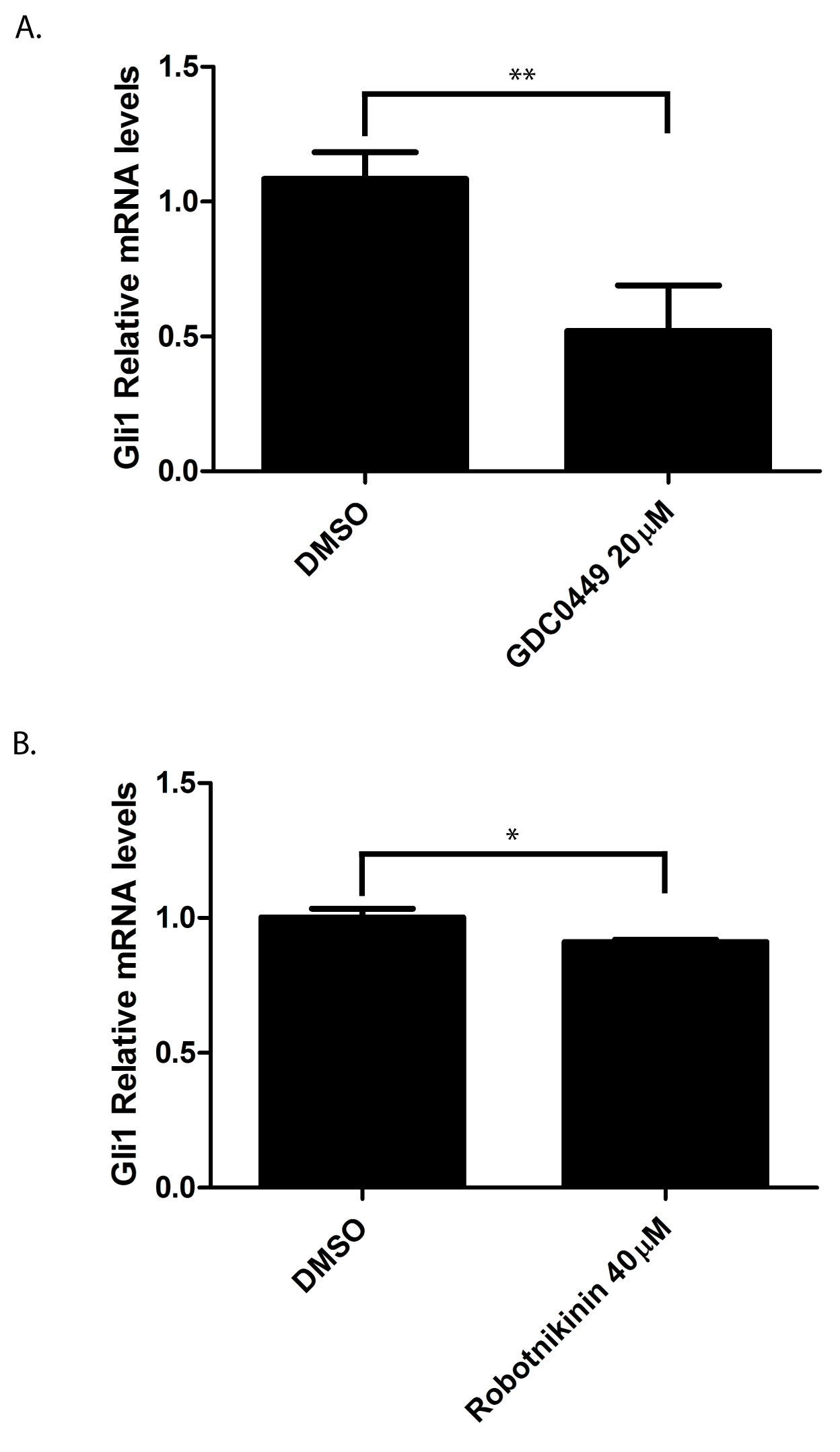

Supplement: Figure S4 — Smo and Shh inhibition by small molecule inhibitors in PANC1 cells reduces Shh signaling. PANC1 cells were treated with either the Smo inhibitor GDC0449 (A) or the Shh-binding antagonist Robotnikinin (B) or DMSO control. Shh signaling was examined by qPCR using Gli1-specific primers 48 h after treatment. A. GDC0449 treatment significantly reduced Gli1 expression (p = 0.0279). B. Robotnikinin also reduced Gli expression but did not reach significance. These data indicate that autocrine Shh signaling can be inhibited in the PANC1 cells used in this study. Our data are supported by several published studies on PANC1 cells [1]–[3]. Gli1 expression is normalized with GAPDH and compared to DMSO control. Error bar represents the standard error of n = 4 (GDC0449 data) and n = 3 (Robotnikinin data) independent experiments performed in duplicate; data were analysed in Graphpad Prism by a two-tailed unpaired student t-test (**, P<0.05; *, P<0.1). (TIF) [file pone.0089899.s004.tif]

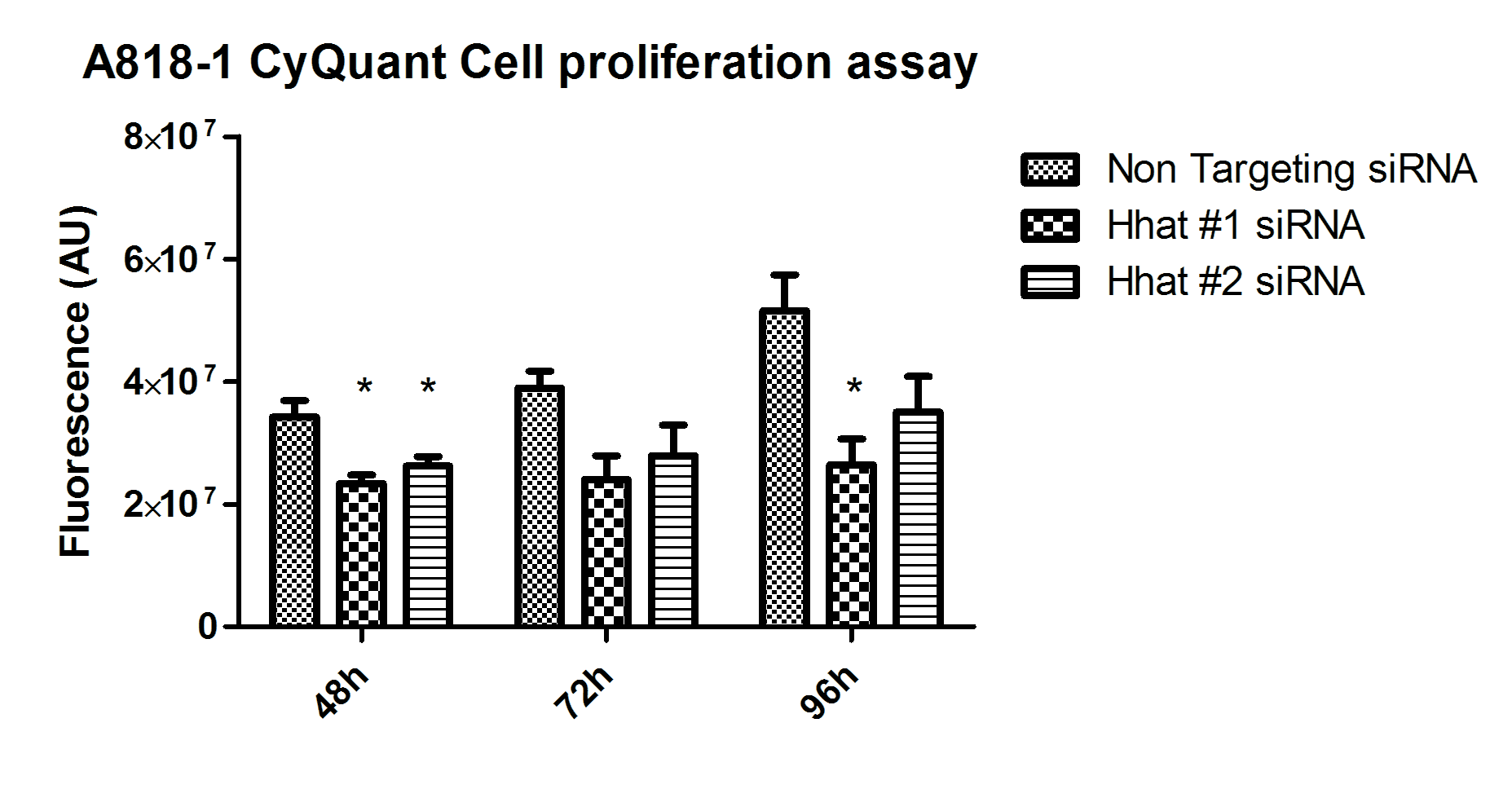

Supplement: Figure S5 — Hhat KD inhibits A818-1 cell proliferation. For each condition, 2×10^4^ A818-1 cells were transfected with the indicated siRNA and then 5×10^3^ cells were plated out in four 96-well plates. Proliferation was subsequently monitored at specific time points by measuring the DNA content using the CyQUANT NF reagent. The Y-axis shows the fluorescence measurements reported as arbitrary units (AU) in each well for the indicated condition. Measurements were made using an Ettan DIGE imager with excitation at 485 nm and emission detection at 530 nm. The experiments were repeated four times, and statistical significance was measured by using the two-tailed t test (*, P<0.05). (TIF) [file pone.0089899.s005.tif]
