# Supplementary material for: Attenuation of Hedgehog Acyltransferase-Catalyzed Sonic Hedgehog Palmitoylation Causes Reduced Signaling, Proliferation and Invasiveness of Human Carcinoma Cells
Source: PLoS One. 2014 Mar 7;9(3):e89899. doi: 10.1371/journal.pone.0089899 (PMC3946499; doi:10.1371/journal.pone.0089899)
Supplement: File S1 — (DOCX) [file pone.0089899.s006.docx]

**Supporting Information**

**Cloning of Recombinant Hhat.** Human Hhat cDNA (Accession No: BC117130) was cloned into pEGFP-N3 mammalian expression vector at the XhoI and BamHI sites HA-Hhat-V5 and Hhat-V5 were created using Gateway® cloning (Invitrogen) into mammalian expression vector pcDNA-DEST40. Entry vectors were made by topoisomerase cloning of PCR inserts into vector pENTR⁄D-TOPO using pENTR directional TOPO cloning kit. PCR inserts were amplified from human Hhat cDNA (Accession No: BC117130) using forward primer 5’-CACCATGTACCCCTACGACGTGCCCGACTACGCCCTGCCCCGATGGGAACTG-3’ or 5’-CACCATGCTGCCCCGATGG-3’ and reverse primer 5’-GTCCGTGGCGTAGGTCTG-3’. Entry vector pENTR⁄D-TOPO containing Hhat constructs and destination vector pcDNA-DEST40 were recombined using LR Clonase II enzyme mix to produce HA-Hhat-V5 or Hhat-V5 expressing constructs. Resulting clones were confirmed by DNA sequencing.

**Production of HEK293a cell lines constitutively expressing Shh or Hhat-V5.** HEK293a cells stably expressing full length human Shh or Hhat with a C-terminal V5 tag (Hhat-V5) were produced by transfecting the cells with a pcDNA-DEST40 vector carrying the full length human Shh or Hhat cDNAs. Cells were transfected using FuGENE HD (Promega) following the manufacturer’s instructions. 3x105 HEK293a cells were seeded into a well of a 6-well plate in DMEM supplemented with 10% FBS. The next day, 2 μg of pcDNA-DEST40-hShh or pcDNA-DEST40-Hhat-V5 were diluted in 100 μl Opti-MEM (Invitrogen), followed by the addition of 5 μl of FuGENE HD. The mixture was incubated at RT for 15 min and then added dropwise to the seeded cells. After 24 h, the cells were trypsinised and seeded into a 10cm plate in selection medium (DMEM + 10% FBS + 500 μg/ml of G418) and incubated at 37oC for 10 days (regularly changing the selection medium). Stable colonies were ring cloned and cultured separately until they were abundant enough to check the Shh or Hhat-V5 expression by immunoblotting.

**Immunoblotting.** Immunoblotting was carried out 24 h after transfection of HEK293 cells with expression vector for Hhat-EGFP. For SDS-PAGE, 0.4 million cells were harvested and lysed by the addition of loading buffer (45 mM Tris (pH 6.8), 10% glycerol, 1% SDS, 20 mM DTT, 0.01% bromophenol blue) followed by syringing. After electrophoresis, proteins were transferred to nitrocellulose membranes (Millipore, Bedford, MA, USA), blocked at room temperature for 1 h in phosphate-buffered saline (PBS) with 5 % skimmed milk, and then incubated with primary antibody for 16 h at 4°C. Goat polyclonal anti-Patched (ab51983) and mouse monoclonal α-Tubulin (DM1A, ab49928) were purchased from Abcam. Rabbit polyclonal anti-GLI-1 (H-300, sc-20687) and rabbit polyclonal anti-Shh (H-160, sc-9024) were purchased from Santa Cruz Biotechnology. Mouse monoclonal anti-GFP was from Roche. Secondary antibodies were horseradish peroxidase (HRP)-conjugated goat anti-mouse immunoglobulin IgG1, HRP-conjugated goat anti-rabbit IgG, and HRP-conjugated donkey anti-goat IgG (used at 1:20,000; Southern Biotech). Bound immunocomplexes were detected using enhanced chemiluminescence detection reagents (Pierce) and were visualized by exposing the membrane to x-ray film (Fuji Super RX), or with ECL Plus reagent and the Ettan DIGE Imager (GE Healthcare).

**Neutravidin Pull-down of Clicked proteins.** Click chemistry samples were prepared as indicated in the methods section, following attachment of the azido-TAMRA-biotin capture reagent, 50µg of protein lysate were incubated for 2h with shaking at room temperature with 20µl of Neutravidin-coated Sepharose beads (Thermo-Fisher, UK). Following the incubation period, beads were pelleted, and the unbound proteins were collected. Beads were then washed 5 times in PBS containing 0.1%SDS and 1% Triton X-100, before being boiled in 1x reducing LDS sample buffer (Invitrogen). Samples were subsequently analyzed by SDS-PAGE and Western blotting, as indicated in the methods section.

**siRNA and plasmid transfections.** Human Hhat siRNA duplex oligomers Hhat-#1 (sense strand 5’-UUAAUCAGGUAUGUGUACAUUCCAGUG-3’) were designed and ordered from MWG Biotech. Mutated Hhat siRNA duplex oligomers (5’-UUAAUCAGGCAUAUGUACGUUCCAGUG-3’) were from Invitrogen. ON-TARGET plus siRNA against human Hhat (5’-AGGACAGUCUGGCCCGAUA-3’; Hhat-#2) and ON-TARGET plus Non-targeting siRNA pool of four non-targeting siRNAs (D-001810-10-05), were from Dharmacon (Thermo Scientific Dharmacon; Epsom, UK). Another negative control applied in this study was SilencerR Negative Control #1 siRNA (Hhat-Scr, Ambion). Transfections were carried out by plating 0.3 million cells per well in a 6-well plate and 6 h later treated with 20 nM siRNA oligomers and 3 μl FuGENE 6 Transfection Reagent (Roche) per reaction. Alternatively, siRNA transfections were carried out using the Metafectene SI reagent (Biontex, Martinsried/Planegg, Germany) according to the manufacturer’s instructions. 30 pmol RNA and 1 μl Metafectene SI reagent were diluted in 30 μl 1x Metafectene SI buffer and allowed to complex for 20 min at room temperature (RT) in a well of a 48-well plate. In the meantime, cells were trypsinised and diluted to 0.8 x 105 cells/ml in complete medium. Suspended cells (250 μl) were added to each well and plates were shaken to ensure even distribution of the cells and reagents. Cells were assayed 72 h post-transfection.

**Semiquantitative Reverse Transcription-PCR (RT-PCR) and qPCR.** Total RNA extraction from cultured cells was done with Trizol (Invitrogen). cDNA was synthesized by random priming from 1 μg of total RNA with SuperScript II Reverse Transcriptase kit (Invitrogen), according to the manufacturer’s instructions. Alternatively, the cDNA was synthesized by random priming from 1 μg of total RNA with the GoScript reverse transcriptase kit (Promega). The following primers were used for the subsequent PCR: human GAPDH (sense, 5’-TTCATTGACCTCAACTACAT-3’; antisense, 5’-GTGGCAGTGATGGCATGGAC-3’); human β-actin (sense, 5’-ATGGATGAGGATATCGCTGCG-3’; antisense, 5’-CTAGAAGCATTTGCGGTGCAC-3’); human Shh (sense, 5’-CGCACGGGGACAGCTCGGAAGT-3’; antisense, 5’-CTGCGCGGCCCTCGTAGTGC-3’) [ref 26 in main text]; human Ptch (sense, 5’-GGTGGCACAGTCAAGAACA-3’; antisense, 5’-ACCAAGAGCGAGAAATGG-3’) [ref 26 in main text]; human Smo (sense, 5’-TTACCTTCAGCTGCCACTTCTACG-3’; antisense, 5’-GCCTTGGCAATCATCTTGCTCTTC-3’) [ref 27 in main text]; human Gli1 (sense, 5’-TTCCTACCAGAGTCCCAAGT-3’; antisense, 5’-CCCTATGTGAAGCCCTATTT-3’) [ref 27 in main text].

Polymerase chain reaction (PCR) with Taq DNA Polymerase (Invitrogen) followed the manufacturer’s instructions. Cycling conditions were: 30 cycles of 30 seconds at 95°C, 30 seconds at 60°C, and 2 minutes/kb at 72°C. PCR products were resolved by electrophoresis on 1.7% agarose gels and visualized by ethidium bromide staining.

Hhat knockdown in the siRNA-treated cells was further validated by qPCR, for which we used the GoTaq qPCR master mix (Promega). Specifically, cDNA from 15 ng input RNA was used in a 20 μl reaction in a 96-well thermal plate, in triplicate. The plates were sealed and run in an ABI 7500 Fast Real-Time PCR System cycler (Applied Biosystems, Life Technologies). Cycling conditions: 40-45 cycles of 95oC for 45 sec, 60oC for 30 sec. Data were analyzed using the ΔΔCt method for determination of relative gene expression by normalization to an internal control gene (GAPDH) and fold expression change was determined compared to a control sample treated with non-targeting siRNA.

**Small molecule inhibition of Smo and Shh.** 1X 105 PANC1 cells were plated out in 12-well plates and allowed to adhere. The following day, the culture medium was substituted for DMEM containing 0.5% FBS and 20µM GDC0449 (Stratech Scientific, Suffolk, UK), 40µM Robotnikinin (Cambridge bioscience, UK) or 0.2% DMSO; media and drugs were renewed every 24h. Total RNA was extracted at 48h and cDNA prepared as indicated in the methods section. Gli1 expression was subsequently determined by qPCR using Gli1 specific primers [4] (forward primer: CAGGGAGTGCAGCCAATACAG, reverse primer: GAGCGGCGGCTGACAGTATA) and data were analyzed using the ΔΔCt method for determination of relative gene expression by normalization to an internal control gene (GAPDH) and fold expression change was determined compared to the DMSO control sample.

**Immunostaining and fluorescence microscopy.** PANC1 cells were seeded onto 6-well plates at a density of 1X105 cells/well and transfected with Hhat-EGFP. 48 h after transfection, the cells were fixed with 4% paraformaldehyde (PFA). Alternatively, 6X104 HEK293a cells stably expressing Hhat-V5 were seeded onto glass coverslips in 24-well plates. 24 h after plating out, cells were fixed with 3% PFA in 1X PBS. Imaging was performed using a Zeiss LSM510 laser-scanning confocal microscope in the FILM imaging facility. Hhat was visualized with mouse monoclonal anti-V5 IgG2A (1:200, Invitrogen) followed by Alexa488-conjugated anti-mouse IgG2A secondary antibody. Golgi complex was visualized after staining with mouse monoclonal anti-GM130 IgG1 (1:600, BD Transduction Laboratories™), and ER with mouse monoclonal anti-protein disulfide isomerase (PDI) IgG1 (1:100, BD Transduction Laboratories™) or anti-calnexin (AF18, 1:50, Sigma) respectively, followed by Alexa568 or Alexa633-conjugated anti-mouse IgG1 secondary antibodies.

**Alkaline phosphatase** (ALP) activity was measured using p-nitrophenyl phosphate substrate. 5 mg p-nitrophenyl phosphate (Sigma) were dissolved in 5 ml ALP reagent buffer (1 M diethanolamine buffer pH 9.8, 0.5 mM MgCl2). 200 µl of ALP substrate solution were added per well of lysed cells and the reaction was incubated at room temperature for 1-2 h. Reaction was stopped by addition of 50 µl 3 M NaOH and absorbance was measured at 405 nm.

**Statistical analysis.** All experiments were replicated at least three times, and statistical significance was measured by using the two-tailed t test. A p value of less than 0.05 was considered to indicate statistical significance. All signals from immunoblots and dot blots were quantified using Scion Image software.

**References:**

1. Singh BN, Fu J, Srivastava RK, Shankar S (2011) Hedgehog Signaling Antagonist GDC-0449 (Vismodegib) Inhibits Pancreatic Cancer Stem Cell Characteristics: Molecular Mechanisms. PLoS ONE 6: e27306.
2. Steg A, Amm HM, Novak Z, Frost AR, Johnson MR (2010) Gli3 mediates cell survival and sensitivity to cyclopamine in pancreatic cancer. Cancer Biology & Therapy 10: 893-902.
3. Strand MF, Wilson SR, Dembinski JL, Holsworth DD, Khvat A, et al. (2011) A Novel Synthetic Smoothened Antagonist Transiently Inhibits Pancreatic Adenocarcinoma Xenografts in a Mouse Model. PLoS ONE 6: e19904.
4. Vestergaard J, Pedersen MW, Pedersen N, Ensinger C, Tumer Z, et al. (2006) Hedgehog signaling in small-cell lung cancer: frequent in vivo but a rare event in vitro. Lung Cancer 52: 281-90.
